# Significant Microsynteny with New Evolutionary Highlights Is Detected through Comparative Genomic Sequence Analysis of Maize CCCH IX Gene Subfamily

**DOI:** 10.1155/2015/824287

**Published:** 2015-10-11

**Authors:** Wei-Jun Chen, Yang Zhao, Xiao-Jian Peng, Qing Dong, Jing Jin, Wei Zhou, Bei-Jiu Cheng, Qing Ma

**Affiliations:** Key Laboratory of Crop Biology of Anhui Province, School of Life Sciences, Anhui Agricultural University, Hefei, Anhui 230036, China

## Abstract

CCCH zinc finger proteins, which are characterized by the presence of three cysteine residues and one histidine residue, play important roles in RNA processing in plants. Subfamily IX CCCH proteins were recently shown to function in stress tolerances. In this study, we analyzed CCCH IX genes in *Zea mays, Oryza sativa*, and *Sorghum bicolor*. These genes, which are almost intronless, were divided into four groups based on phylogenetic analysis. Microsynteny analysis revealed microsynteny in regions of some gene pairs, indicating that segmental duplication has played an important role in the expansion of this gene family. In addition, we calculated the dates of duplication by Ks analysis, finding that all microsynteny blocks were formed after the monocot-eudicot divergence. We found that deletions, multiplications, and inversions were shown to have occurred over the course of evolution. Moreover, the Ka/Ks ratios indicated that the genes in these three grass species are under strong purifying selection. Finally, we investigated the evolutionary patterns of some gene pairs conferring tolerance to abiotic stress, laying the foundation for future functional studies of these transcription factors.

## 1. Introduction

Transcription factors (TFs) are critical regulators of gene expression that control many important biological processes, such as cellular morphogenesis, signal transduction, and environmental stress responses [1]. Zinc finger TFs belong to one of the largest TF families in plants and can be categorized into at least 14 families, such as RING finger, WRKY, DOF, and LIM families [2–4]. These TFs have been proven to regulate gene expression with the aid of DNA-binding or protein-binding proteins. However, previous reports discovered a new type of* Arabidopsis* zinc finger proteins, named CCCH zinc finger family that is involved in mRNA binding and processing [5]. CCCH-type proteins are TFs with a typical motif consisting of three cysteine residues and one histidine residue. CCCH proteins, containing one to six copies of CCCH-type zinc finger motifs, were originally defined as C-X6-14-C-X4-5-C-X3-H, but a recent study has redefined them as C-X4-15-C-X4-6-C-X3-H, following genome-wide analysis of rice and* Arabidopsis thaliana* CCCH proteins [5].

Recent studies have revealed that CCCH proteins participate in the regulation of plant growth, developmental processes, and environmental responses. In rice, a novel nuclear-localized CCCH-type zinc finger protein, OsDOS, is involved in delaying leaf senescence by integrating developmental cues to the jasmonic pathway [6]. In pepper and rice, the CCCH-domain proteins CaKR1 and OsC3H12 were shown to protect plants from bacterial blight [7, 8]. During senescence in* Arabidopsis*, HUA1, a CCCH-type zinc finger protein with six tandem CCCH motifs, likely participates in regulating flower development [9]. In addition, some CCCH zinc finger proteins are also involved in the abiotic stress response. Two closely related proteins in* Arabidopsis*, AtSZF1 and AtSZF2, are both involved in modulating salt stress tolerance in plants [10]. Recently,* ZmC3H4* and* ZmC3H28*, which are indirectly regulated by ABA or drought, and 10 other maize CCCH IX genes were found to be responsive to abiotic stress [11].

The CCCH-type zinc finger protein family had been studied in some model organisms on a phylogenetic scale, but its particular evolutionary pathway is still poorly understood. Gramineae, which evolved approximately 60–70 mya (million years ago) from a common ancestor, includes a number of agronomically important crops, such as* Oryza sativa*,* Zea mays*, and* Sorghum bicolor* [12]. The origin of these crops dates back to approximately 50 to 65 mya, and the family has now expanded to over 10,000 species [13]. Whole-genome analyses have revealed high levels of genetic conservation in grasses over the course of evolution, yet these studies have revealed no trace of microsynteny (preservation of a specific local gene order) across grasses [14, 15]. The goal of this study was to identify stress-responsive genes in* Z. mays*,* O. sativa*, and* S. bicolor* and to analyze the evolutionary relationships of CCCH IX subfamily at the molecular level using microsynteny analysis. Specifically, we searched for CCCH IX subfamily genes and predicted their structures. To determine the expression patterns of CCCH IX genes in maize tissue, we utilized publically available microarray data from Sekhon et al. [16]. The expression map was shown in Figure S1 (in Supplementary Material available online at http://dx.doi.org/10.1155/2015/824287). In addition, we identified duplication events and calculated Ka/Ks values. Finally, we designed microsynteny maps to identified conservative CCCH IX genes during evolution. The results of this study lay the foundation for future functional studies of CCCH IX subfamily genes.

## 2. Materials and Methods

### 2.1. Identification of Genes Encoding CCCH IX Proteins

The recent versions of genome, protein, and cDNA sequences for the following three grass species were downloaded from the respective genome sequence sites:* Oryza sativa* (version 7.0) from the Rice Genome Annotation Project (http://rice.plantbiology.msu.edu/),* Zea mays* (version 2.0) from the B73 Maize Genome Project (http://www.maizesequence.org/index.html), and* Sorghum bicolor* (version 1.0) from the DOE-JGI Community Sequencing Program (CSP) (http://www.phytozome.net/sorghum.php). These nucleotide and protein sequences were used to build local databases using DNATOOLS software [17]. The conserved CCCH domain (PF00642) based on the Hidden Markov Model (HMM) was obtained from http://pfam.sanger.ac.uk/ (Pfam database) [18]. This HMM profile was used as a query to search against the protein database with the BLASTp program (version blast-2.2.9-ia32-win32) (*p* value = 0.001). This step was crucial for finding as many similar sequences as possible. All predicted protein sequences of genes were analyzed in the Pfam HMM database and the SMART tool (http://smart.embl-heidelberg.de/) to identify CCCH domains, and proteins without these regions were excluded from the dataset [19]. All potential sequences were aligned using Clustal W, and all identical sequences were checked manually to remove redundant genes prior to subsequent analysis [20].

The molecular weight (kDa) and isoelectric point (pI) of each gene were calculated using the online ExPASy tools (http://www.expasy.org/tools/) [21]. The intron distribution patterns and intron/exon boundaries of the CCCH IX genes were deduced by using Gene Structure Display Server (http://gsds.cbi.pku.edu.cn/) to compare the predicted full-length cDNA or coding sequences with the corresponding genomic sequences [22].

### 2.2. Phylogenetic Analysis of CCCH IX Genes

The phylogeny of CCCH IX genes was performed by clustering and aligning the protein sequences using Clustal W. The phylogenetic tree was constructed using MEGA 6.0 by the neighbor-joining (NJ) method with the following parameters: Poisson correction, pairwise deletion, and bootstrapping (1,000 replicates) [23]. To confirm the robustness of the NJ tree, an ML tree was constructed using the maximum likelihood method (MEGA 6.0; bootstrap = 1,000 replicates, amino acid substitution model, and Jones-Taylor-Thornton matrix), and an MP tree was constructed using the maximum parsimony method (MEGA 6.0; bootstrap = 1,000 replicates).

### 2.3. Detection of CCCH IX Gene Expansion

The following analysis was performed to obtain in-depth knowledge of the evolutionary relationships among CCCH IX genes and to determine whether these genes were derived from segmental duplication or tandem duplication events. Tandem duplication is characterized as the presence of multiple gene family members within the same or neighboring intergenic regions. To be defined as a segmental duplication event, each pair of protein-coding genes (excluding noncoding RNA genes, pseudogenes, and so on) in each genome must reside within a duplicated block; moreover, there must be a high similarity between their neighboring protein-coding genes at the amino acid level [24]. First, all identified CCCH IX genes were used as the original anchor points. Next, 100 kb sequences upstream and downstream of each anchor point were compared by pairwise BLASTp (*E*-value ≤ 10^−10^) analysis to identify duplicated genes between two independent regions. The number of protein-coding genes flanking any anchor point was then counted [25]. When three or more such genes pairs with syntenic relationships were identified in two regions, the regions were considered to have been derived from a large-scale duplication event [26, 27].

### 2.4. Microsynteny Analysis

Microsynteny analysis across the three species was carried out based on comparisons of the specific regions containing CCCH IX genes. Levels of similarity between the flanking genes of each CCCH IX gene in one species and those in the other species were determined by pairwise comparisons using the BLASTp program. A syntenic block was defined as a region where three or more conserved homologs (BLASTp *E*-value ≤ 10^−20^) were located within a 100 kb region between genomes [28, 29]. The relative syntenic quality in a region was calculated based on the sum of the total number of genes in both conserved sequence regions, excluding tandem duplication. A circular microsynteny map was also constructed using the program Circos-0.54, which utilizes the Perl language [30].

### 2.5. Ks Analysis of Homologous Segments

The time of divergence of duplicated gene pairs within each duplicated block or the divergence of homologous segments was estimated by calculating Ks values between homologous genes using DnaSP (version 5.10) [31–33]. Sliding window analysis of nonsynonymous substitutions per nonsynonymous site (Ka/Ks) ratios was conducted with the following parameters: window size, 150 bp; step size, 9 bp [34].

Ks values can also be used to calculate the timing of large-scale replications. For each pair of duplicated regions, the mean Ks value for individual homologs in flanking conserved genes was calculated and used to determine the approximate time of divergence. Hence, Ks could be converted to the divergence time beyond the Gramineae evolutionary rate of each locus. The divergence time (*T*) was calculated as *T* = Ks/(2 × 6.5 × 10^−9^) × 10^−6^ mya [35].

## 3. Results

### 3.1. Phylogenetic and Sequence Structure Analysis of CCCH IX

**Figure 1:**
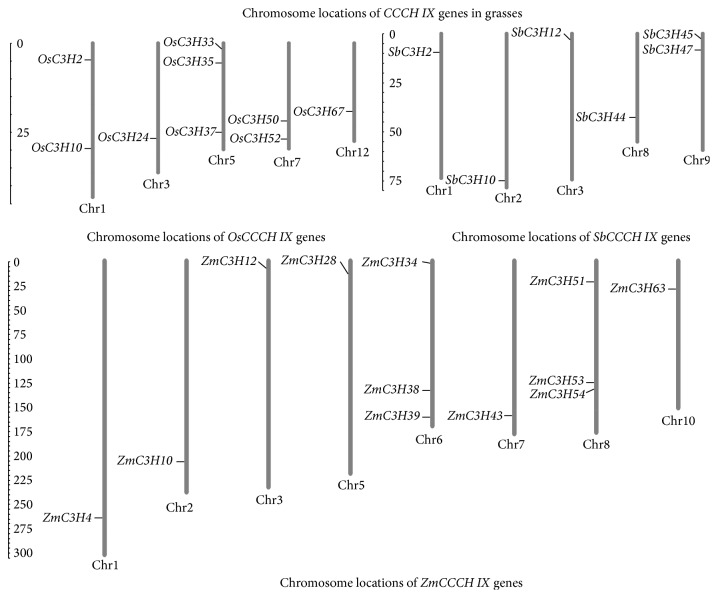
Chromosomal locations of CCCH IX genes in three Gramineae species (*O. sativa*,* S. bicolor,* and* Z. mays*). The 27 CCCH IX genes are randomly distributed on chromosomes, including nine rice genes, six sorghum genes, and twelve maize genes.

In the previous study, 67 CCCH genes were identified in* Z. mays*. Then, we identified 55 genes encoding CCCH zinc finger proteins in* S. bicolor* (Supplementary Table 1) using the BLASTp program. For convenience, we assigned names to these genes (*SbC3H1–SbC3H55*) according to their chromosomal positions. Based on previous studies, we identified six* S. bicolor* genes in the CCCH IX subfamily [5, 11]. A total of 27 CCCH IX genes, which are listed in Table 1 (nine genes from* O. sativa,* six genes from* S. bicolor*, and twelve genes from* Z. mays*), were subjected to further analysis. The lengths of the 27 encoded CCCH IX proteins vary from 225 to 764 aa, with an average of 476 aa. Other pieces of information, including the clone number, chromosomal location, molecular weight (Mw), and isoelectric point (pI) of each CCCH IX gene/protein, are listed in Table 1. To determine the organization and distribution of CCCH IX genes on different chromosomes, we constructed a chromosome map. The 27 CCCH IX genes are randomly distributed on chromosomes, as shown in Figure 1.

**Figure 2:**
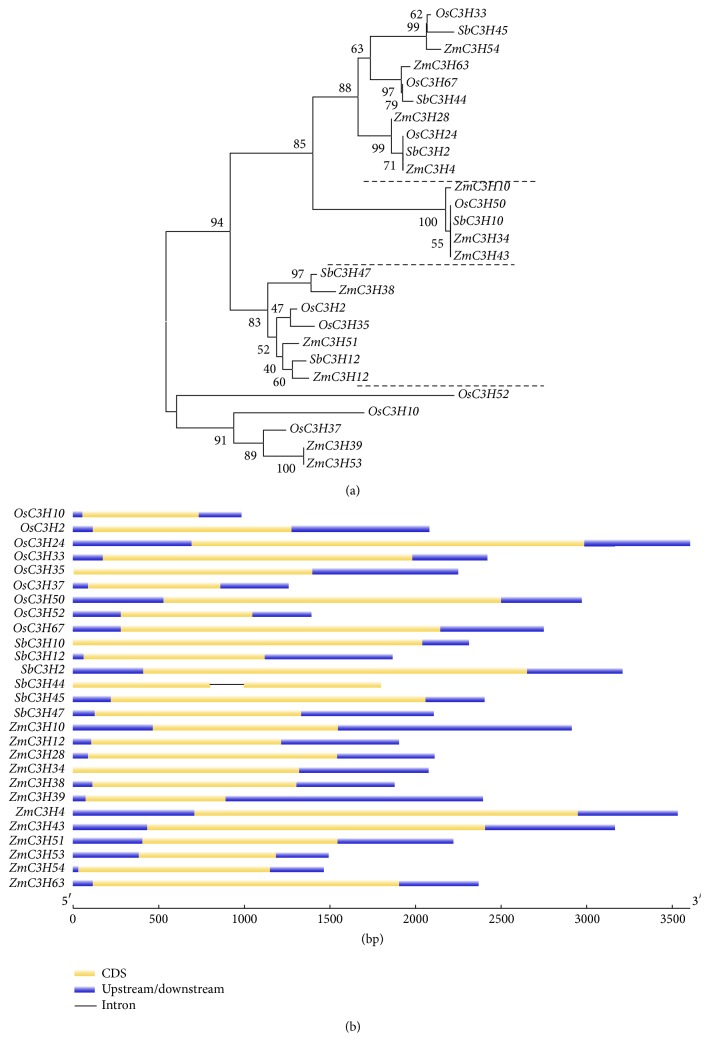
(a) Phylogenetic tree of CCCH IX proteins from* O. sativa*,* S. bicolor*, and* Z. mays*. This tree was constructed using the MEGA 6.0 program by the N-J method with 1,000 bootstrap replicates based on amino acid sequence. The tree is divided into four clades (clades I–IV). (b) Exon-intron structures of 27 CCCH IX genes in three species. Exons and introns are indicated by green thick lines and thin gray lines, respectively. The untranslated regions (UTRs) are indicated by blue lines.

**Figure 3:**
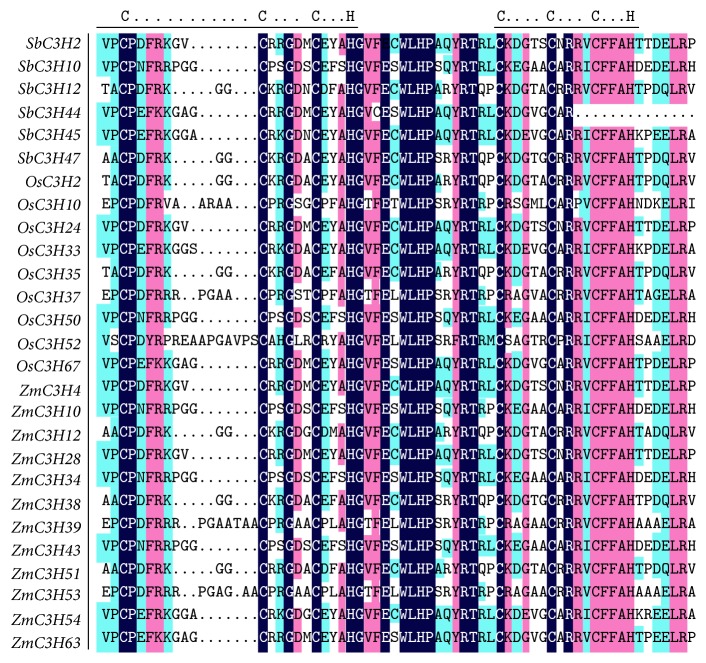
Alignment of the amino acid sequences of CCCH IX proteins in three grass species. Identical (100%), conservative (75–99%), and blocks (50–74%) of similar amino acid residues are shaded in deep blue, dark pink, and light blue, respectively. The conserved CCCH zinc finger motifs are indicated by straight lines.

To explore the evolutionary relationships between members of the CCCH IX zinc finger subfamily, we constructed a phylogenetic tree using the neighbor-joining (NJ) method based on protein sequence alignment. The phylogenetic tree is divided into four clades (Figure 2(a)). Although different clades have different numbers of members, clades 1–3 consist of proteins from all three grass species, whereas* S. bicolor* proteins are absented from clade 4. These differences may have been derived from partial gene loss that may have occurred after large-scale duplication events after the formation of new species, which drove species separation. The phylogenetic relationships depicted in the ML and MP trees are largely consistent with these results (Figure S2). We investigated the sequence structure by exon-intron structure analysis (Figure 2(b)) (http://gsds.cbi.pku.edu.cn/) [36]. The most closely related CCCH IX members in the same clades share similar gene lengths and exon lengths. The only exception was observed in the sequence (*SbC3H44*) from* S. bicolor* that contained one intron and one CCCH domain (Figure 3). Interestingly, the remaining 26 genes are entirely intronless, and they contain two CCCH domains without exception. These findings imply that this subfamily of genes has retained sequences, which have been conserved at the structural level, including exon-intron structure and the number of CCCH domains, throughout millions of years of evolution.

### 3.2. Complicated Duplication Events Have Contributed to CCCH IX Expansion

**Figure 4:**
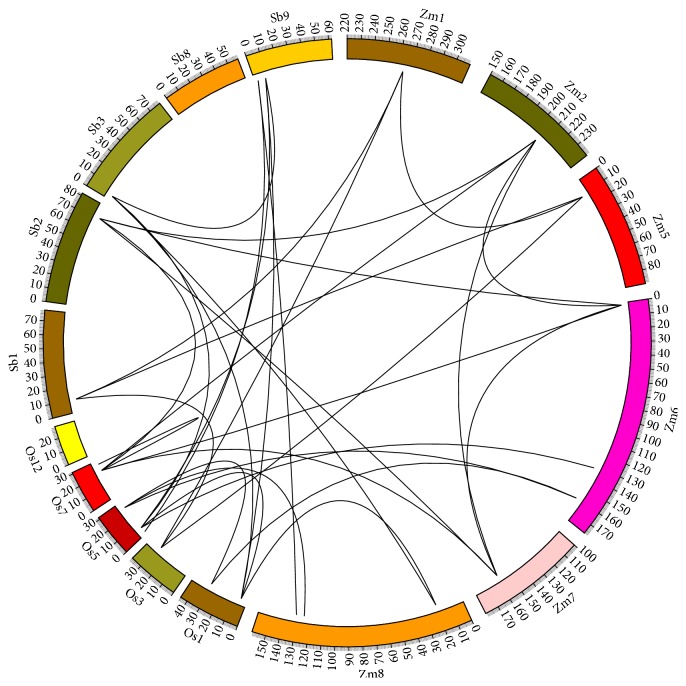
Extensive microsynteny of CCCH regions across* O. sativa*,* S. bicolor*, and* Z. mays. O. sativa* chromosomes (labeled Os) are indicated by orange boxes.* S. bicolor* and* Z. mays* chromosomes (labeled Sb and Zm, resp.) are shown in blue and green, respectively. CCCH IX regions in the three grasses are shown in the circle. Black lines show syntenic relationships.

**Figure 5:**
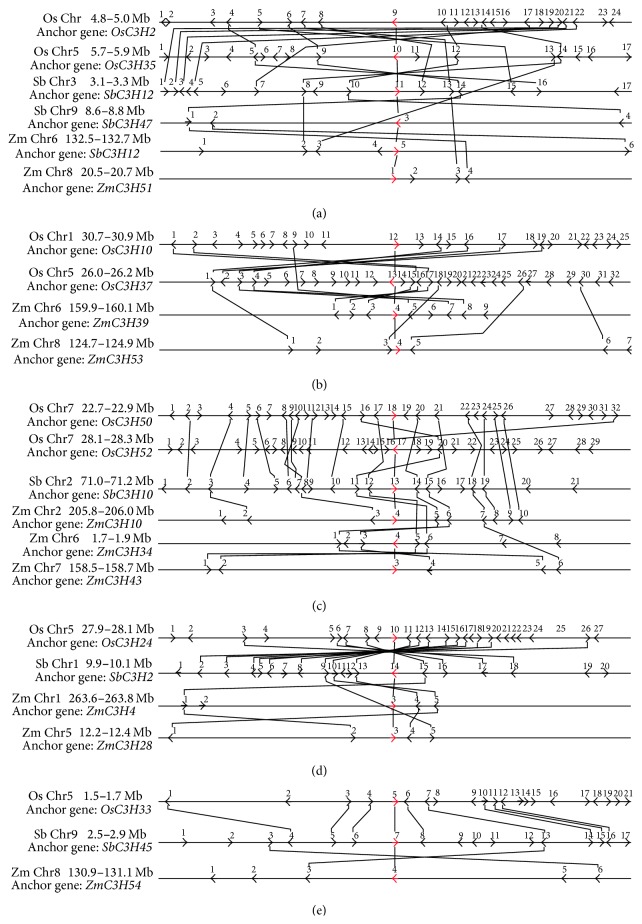
Microsynteny maps of CCCH IX genes in grasses. Red arrows represent anchor (CCCH IX) genes, and upstream and downstream genes are represented by black arrows. All genes are numbered from left to right for each segment. Black lines connect conserved gene pairs.

We estimated the chromosomal locations of CCCH IX genes in these grasses and examined the evolutionary relationships between these genes. However, in the relevant chromosomes, there was no universal tandem duplication, due to the irregular distribution of CCCH IX genes. To determine whether the regions flanking CCCH IX genes have undergone large-scale duplication events during the evolution, we compared the flanking genes of any two CCCH IX genes. If three or more flanking genes had a best nonself-match according to BLASTp (*E*-value ≤ 10^−10^ within species and *E*-value ≤ 10^−20^ between species), we considered that these members belonged to a duplicated block. Based on this information above, we investigated the evolutionary origins and evolutionary relationships within and between grasses species using a Perl script (Figure 4). Initially, we found 11 duplicated gene segments constituting a network within species, including five maize genes and four rice genes. However, we subsequently identified five groups containing 23 genes (the five groups are shown in Figure 5, with red arrows representing the 23 genes) from species exhibiting tight microsynteny relationships.

In rice, we found four highly similar genes among the sequences flanking both sides of* OsC3H2/OsC3H35* (Figure 5(a)). A duplication event may have occurred during the evolutionary history of this pair of genes. Based on this notion, we reasoned that this pair of genes from one group shares a closer phylogenetic relationship than the others, which helps confirm the results of phylogenetic analysis (Figure 2). In addition, the gene pair* OsC3H10/OsC3H37* is surrounded by six conserved genes compared with the gene pair* OsC3H2/OsC3H35*, and their microsyntenic relationship is closer than that of the latter pair (Figure 5). In addition, the collinear gene pair* OsC3H2/OsC3H35* is located on OsChr1/OsChr5, and the gene pair* OsC3H10/OsC3H37* is present on the same chromosome (Figure 1). These results suggest that, during the evolution of the rice genome, these two chromosome segments were generated by whole-genome duplication; a large-scale duplication has affected the evolution of CCCH IX genes in rice. In sorghum, the gene pair* SbC3H12/SbC3H47* was identified as microsyntenic relationship (Figure 5(a)), because only six CCCH IX genes belong to this subfamily and they exhibit relatively sparse microsynteny. Based on the phylogenetic tree (Figure 2), each branch of* S. bicolor* genes has a high degree of similarity with that of other grasses.

In maize, five CCCH IX genes are located in the duplicated section of the genome, and they share a microsyntenic relationship (Figures 5(c) and 5(d)).* ZmC3H10/ZmC3H34/ZmC3H43* exhibit tight microsynteny. These results suggest that a large-scale duplication event has occurred during the process of evolution of the maize genome. This duplication event can also be deduced by examining the gene pair* ZmC3H4/ZmC3H28.* We compared 100 kb sequences upstream and downstream of each anchor point by pairwise alignment, finding that many duplicated regions occurred as mentioned above in the three species. These results indicate that large-scale duplication events have resulted in the production of paralogous genes throughout evolutionary history.

Microsynteny analysis can be used to predict the locations of homologous genes in different species [37]. Regions with 80% of close homologs in the same order and transcriptional orientation are characterized as exhibiting conserved microsynteny [38]. We used this analysis to deduce the molecular evolutionary origins and orthologous relationships within the chromosome regions containing CCCH IX genes in the three grass species. We performed a stepwise gene-by-gene reciprocal comparison to gauge the linkages between CCCH IX regions. Based on these detailed comparisons and rigorous analysis, the results of mapping of conserved microsynteny regions are similar to those shown in the phylogenetic tree, but analysis of the flanking fragments represents a more thorough approach than phylogenetic analysis.

### 3.3. Conserved Microsynteny of CCCH IX Genes between Species

The genes from the three grasses exhibiting tight microsyntenic relationships were divided into five groups (Figure 5). Genome segments in the same group were likely derived from a single sequence during evolution, which resulted in species differentiation. Sequence segments from the same group are considered to be homologous genes whereby genetic evolution led to species separation. In group (a), we observed a marked opposite-direction microsynteny relationship among* OsC3H2/OsC3H35, SbC3H12/SbC3H47*, and* ZmC3H38/ZmC3H51* segments (Figure 5). In this group, these gene fragments were derived from a duplication event and are orthologous to each other. The same situation exists in group (b), where we observed microsynteny in a series of genes:* OsC3H10*,* OsC3H37*,* ZmC3H39*, and* ZmC3H53* (Figure 5). Chromosome inversions result in reversals in gene order. Group (c) exhibited a higher level of compact microsynteny, especially* OsC3H50*,* SbC3H10*,* ZmC3H10*, and* ZmC3H43* (Figure 5). On the contrary,* SbC3H10*,* ZmC3H34*, and* ZmC3H43* exhibited opposite-orientation microsynteny. In group (d),* OsC3H24*,* SbC3H2*,* ZmC3H4*, and* ZmC3H28* appeared microsyntenic, especially the pair* OsC3H24* and* SbC3H2*, which has nine best matching genes according to BLASTp alignment (Figure 5). Group (e) genes* OsC3H33* and* SbC3H45* were identified as having successive same-direction microsynteny (Figure 5). In CCCH IX regions in these species, the chromosomal composition of one species is often assembled from two or more successive segments. For example, fragment SbChr3 appears to match OsChr1 and OsChr5, and fragment SbChr1 appears to match OsChr3 and ZmChr1. In the CCCH IX subfamily, chromosomal translocation is a common phenomenon that occurs during the process of differentiation. Based on previous study (in which 12 stress-responsive CCCH IX genes were identified in maize [11]),* OsC3H24*,* SbC3H2*,* SbC3H45*, and so on (as mentioned above) may be involved in regulating abiotic stress through microsynteny mapping, as shown in Figure 5.

### 3.4. Estimating the Dates of Duplication Events

**Figure 6:**
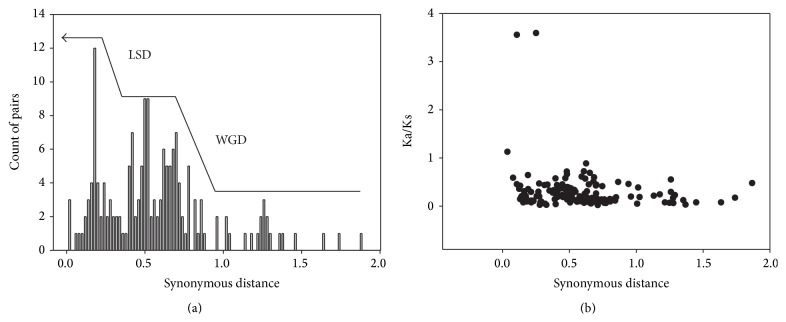
(a) Distribution of synonymous distance (Ks) between paralogous genes flanking duplicated CCCH IX genes in the three grass species. The histogram depicts the number of duplicate gene pairs (*y*-axis) versus synonymous distance between pairs (*x*-axis). CCCH IX blocks experienced whole-genome duplication (WGD) in the first stage of their evolution and large-scale duplication (LSD) in the second stage. (b) Ka/Ks ratios of duplicated CCCH IX genes and their flanking paralogs in the three grasses. The *y*- and *x*-axes denote the Ka/Ks ratio and synonymous distance for each pair, respectively.

Based on the assumption that the synonymous mutation rate at each site is stable over time [39], we calculated the duplication event date based on the conserved flanking protein-coding genes. Each pair of proteins in a microsynteny block was aligned at the amino acid level, and codons from gapless aligned regions were used to calculate Ks values using CodeML [24]. We removed any Ks values > 2.0 due to the risk of saturation [40]. The approximate date of the duplication event was then calculated using the mean Ks and an estimated rate of silent site substitutions of 6.5 × 10^−9^ substitutions/synonymous site/year. The divergence time (*T*) was calculated as *T* = Ks/(2 × 6.5 × 10^−9^) × 10^−6^ mya [35]. The mean Ks values for each duplication event and the estimated dates are listed in Table 2. The results reveal that, for these duplication events, few genes and their flanking fragments expanded before Gramineae speciation (60–70 mya) [40]. The subsequent whole-genome duplication played an important role in the expansion of genes containing CCCH IX regions, leading to complete genome diploidization along with gene rearrangement and loss. The related gene duplication events occurred frequently, leading to further integration of these genes in maize and sorghum approximately 15 mya (Figure 6(a)). We identified the same pattern for synchronous replication during evolutionary history, which helps confirm that section is synchronous in function.

### 3.5. Selection Pressure on CCCH IX Genes

Since large-scale duplication has contributed to genome evolution, we also calculated the selection pressure among CCCH IX duplicated genes [41]. We calculated the Ka/Ks ratios for 31 pairs of conservative homogenous genes, along with their flanking segments. We found that the Ka/Ks ratios of homologous replication groups were less than 1, except for those of two flanking genes (Figure 6(b)), suggesting that these genes were subjected to purifying selection over the course of evolution [42]. To investigate the selection pressure on these genes in distinct regions, we performed sliding window analysis of Ka/Ks ratios using the following parameters: window size, 150 bp; step size, 9 bp. The Ka/Ks values reveal that the selection pressure differed among sites with sequence differences. We detected the stronger purifying selection in the CCCH domain, except for* ZmC3H10/ZmC3H34* and* SbC3H45/ZmC3H54* (Figure S3). The Ka/Ks ratios of most sequences were < 1 suggesting that these gene pairs evolved under purifying selection. Purifying selection can remove detrimental mutations and has probably made the CCCH IX sequences consistent across evolutionary history. Hence, the CCCH IX genes are important for plant growth and development.

## 4. Discussion

CCCH IX genes are thought to play a variety of roles in plant growth, development, and stress resistance [6, 9, 10]. In this study, we selected 27 abiotic stress-responsive CCCH IX genes in* Z. mays*,* O. sativa,* and* S. bicolor* based on phylogenetic tree analysis and their genetic structures, as described in previous reports [5, 11]. The CCCH IX subfamily was characterized into four classes based on our interspecific phylogenetic tree (Figure 2). We determined that these specific genes are almost intronless and that they respond to various adverse environmental factors throughout the plant's life cycle [43].

By calculating the dates of duplication of homologous segments and examining the phylogenetic tree, we determined that the most recent (15 mya) duplication events likely occurred in maize and sorghum. Thus, maize and sorghum have probably undergone a series of evolutionary events and experienced a higher rate of evolution than rice. We observed strong microsynteny among rice, maize, and sorghum genes, but this process is not simple, as transsituation, inversion, loss, and segmental duplication have occurred to varying degrees, which act as the driving force in evolution. Such a process is necessary for the expansion of gene families over the course of evolution. For example, through the analysis of microsynteny, we identified segmental duplication in OsChr1/OsChr5 and ZmChr6/ZmChr8; such duplication frequently occurs among genes.

The CCCH IX microsynteny maps suggested that these genes have been conserved over the course of evolution. Chromosomes contain many syntenic segments that have undergone transsituation, inversion, deletion, and duplication. The gene order has been retained in syntenic segments. In such segments, key genes can be identified from other closely related species on homologous chromosomes at the same relative locations. In the CCCH IX subfamily,* ZmC3H4/ZmC3H28/ZmC3H54* are stress-response genes, and the expression level of* ZmC3H54* is the highest (Figure S1). Therefore, we can deduce that* OsC3H33*,* SbC3H45*,* OsC3H24*, and* SbC3H2* might be responsive to abiotic stress according to the detailed microsynteny analysis (Figure 5) [11]. This conclusion is supported by the close relationship between ATSZF1, ATSZF2, and CaKR1, which were previously identified as stress-response genes [7, 10]. Our analysis of CCCH IX genes demonstrated that the CCCH IX genes and their flanking protein-coding genes are subjected to purifying selection. Subsequently, we conducted sliding window analysis to detect gene sequences with unusual selection pressure, which provided more insights into the effects of the environment and abiotic stress on this subfamily.

In the current study, comparisons among the genes across the three Gramineae genomic sequences demonstrated that extensive large-scale genome duplication has occurred in the CCCH IX subfamily before the species separated 60–70 mya [39]. CCCH IX genes have undergone dramatic expansion followed by whole-genome duplication, which led to speciation approximately 60 mya (Figure 6(a)). In general, we found that CCCH IX genes evolved through multiple large-scale duplication events, which are similar to the events that have driven the evolution of protein-coding genes, but the structure, order, and transcriptional orientation of the CCCH IX genes have stayed the same. We then analyzed the evolutionary history of CCCH IX genes in the subsequent tens of thousands of years at the molecular level and performed detailed microsynteny analysis of the abiotic-responsive gene pairs. The results of this study provide a foundation for further investigating the molecular evolution and functions of CCCH IX genes, particularly for members with potentially important roles in regulating abiotic stress responses in plants. However, further experiments should be conducted to directly explore the functions of CCCH IX genes.

## 5. Conclusion

In this study, we identified and analyzed stress-responsive members of the conserved CCCH IX subfamily through comparative genomic analysis. Some pairs of regions exhibited microsyntenic relationships during evolution according to microsynteny maps. In addition, we calculated the date of duplication by performing Ks analysis and examining Ka/Ks ratios. Through microsynteny analysis, we investigated the evolutionary patterns of* OsC3H24/OsC3H33* and* SbC3H2/SbC3H45*, which function in the response to abiotic stress. The results of this study lay foundation for future functional analyses of these TFs.

## Supplementary Material

For the big data, we put supplementary figures and tables in Supplementary Material. Supplementary Figure 1 showed expression profiles of CCCH IX genes across different tissues in maize; Supplementary Figure 2 showed phylogenetic relationship of CCCH IX genes constructed by NJ, ML, and MP methods; Supplementary Figure 3 showed sliding window analysis of duplicated CCCH IX genes in three grass species. Supplementary TABLE 3 listed CCCH genes in *Sorghum bicolor*. Circos use steps: give the detailed steps to draw figure 4 by circos-0.54 program.

## Figures and Tables

**Table 1:** The 27 CCCH IX genes identified in three species and their sequence characteristics (gene ID, ORF, MW, PI, and chromosome locations).

Gene name	Gene identifier	ORF (aa)	MW (Da)	pI	Chromosome	Chromosomal localization	
						Star	End
*OsC3H2*	LOC_Os01g09620.1	386	41422.51	6.41	Os1	4949047	4951126
*OsC3H10*	LOC_Os01g53650.1	225	24982.17	6.06	Os1	30824689	30825670
*OsC3H24*	LOC_Os03g49170.1	764	81043.54	6.63	Os3	28008600	28012204
*OsC3H33*	LOC_Os05g03760.1	601	63235.89	8.65	Os5	1662021	1664438
*OsC3H35*	LOC_Os05g10670.1	464	49684.7	9.02	Os5	5846045	5848291
*OsC3H37*	LOC_Os05g45020.1	255	28257.88	5.39	Os5	26171092	26172349
*OsC3H50*	LOC_Os07g38090.1	657	69391.93	6.61	Os7	22840986	22843954
*OsC3H52*	LOC_Os07g47240.1	280	31590.77	8.04	Os7	28233256	28234642
*OsC3H67*	LOC_Os12g33090.1	619	64725.96	6.04	Os12	20018555	20021302
*ZmC3H4*	GRMZM2G180979_P01	746	79771.77	6.4	Zm1	263731420	263734946
*ZmC3H10*	GRMZM2G099622_P03	360	37921.57	6.37	Zm2	205904041	205906953
*ZmC3H12*	GRMZM5G853245_P03	370	39786.59	6.46	Zm3	6681798	6683631
*ZmC3H28*	GRMZM5G845366_P01	482	52092.46	7.97	Zm5	12382780	12384893
*ZmC3H34*	AC233871.1_FGP008	416	45437.96	8.78	Zm6	1823029	1825102
*ZmC3H38*	GRMZM5G801627_P01	394	42104.18	8.02	Zm6	132663265	132665063
*ZmC3H39*	GRMZM2G004795_P01	270	29691.44	5.67	Zm6	160013893	160016282
*ZmC3H43*	GRMZM5G842019_P01	656	69821.35	6.88	Zm7	158677416	158680576
*ZmC3H51*	GRMZM2G173124_P03	378	40178.89	6.46	Zm8	20669510	20671733
*ZmC3H53*	GRMZM2G093404_P01	262	28025.75	6.4	Zm8	124841672	124843159
*ZmC3H54*	GRMZM2G117007_P01	372	40034.27	8.65	Zm8	131041707	131043172
*ZmC3H63*	GRMZM2G027298_P01	594	62657.48	5.71	Zm10	27949011	27951381
*SbC3H2*	SB01G011150	745	79472.39	6.51	Sb1	10009063	10012269
*SbC3H10*	SB02G036710	680	72441.2	6.88	Sb2	71102658	71104964
*SbC3H12*	SB03G003110	350	37662.05	8.2	Sb3	3207828	3209693
*SbC3H44*	SB08G016640	533	56473.43	5.89	Sb8	44663680	44665480
*SbC3H45*	SB09G002390	611	64185.57	8.21	Sb9	2607622	2610024
*SbC3H47*	SB09G006050	399	42664.56	6.21	Sb9	8731871	8733975

Open reading frame (ORF), molecular weight (MW), and isoelectric point (IP).

**Table 2:** Estimation of the dates of large-scale duplication events in three grasses.

Synteny blocks of CCCH IX genes	Number of conserved flanking protein-coding genes	Synonymous sites	Ks (mean ± s.d.)	Date (mya)
*OsC3H2* & *OsC3H35*	5	299.25	1.0927 ± 0.4254	84.0538
*OsC3H2* & *SbC3H47*	3	292.50	1.0915 ± 0.4868	83.9615
*OsC3H2* & *SbC3H12*	13	268.75	0.5470 ± 0.2981	42.0769
*OsC3H2* & *ZmC3H51*	3	293.00	0.7827 ± 0.4078	60.2077
*OsC3H35* & *SbC3H12*	5	274.42	0.9811 ± 0.3190	75.4692
*OsC3H35* & *SbC3H47*	3	296.67	0.4635 ± 0.1314	35.6538
*SbC3H12* & *SbC3H47*	3	271.58	0.8632 ± 0.5594	66.4000
*SbC3H12* & *ZmC3H51*	3	275.17	0.2552 ± 0.0552	19.6308
*OsC3H35* & *ZmC3H38*	3	302.58	0.9811 ± 0.6874	75.4692
*OsC3H10* & *OsC3H37*	7	173.67	0.7084 ± 0.2090	54.4923
*OsC3H10* & *ZmC3H39*	6	169.08	0.6818 ± 0.1138	52.4462
*OsC3H37* & *ZmC3H39*	6	193.00	0.5051 ± 0.0373	38.8538
*OsC3H37* & *ZmC3H53*	5	197.83	0.5696 ± 0.1238	43.8154
*OsC3H50* & *ZmC3H10*	9	289.33	0.5867 ± 0.1815	45.1285
*OsC3H50* & *SbC3H10*	16	515.00	0.5893 ± 0.1738	45.3308
*OsC3H50* & *ZmC3H34*	5	298.42	0.6653 ± 0.1309	51.1769
*OsC3H50* & *ZmC3H43*	6	501.00	0.6014 ± 0.1371	46.2615
*SbC3H10* & *ZmC3H10*	7	292.67	0.1816 ± 0.0469	13.9692
*SbC3H10* & *ZmC3H34*	5	307.92	0.1692 ± 0.0579	13.0154
*SbC3H10* & *ZmC3H43*	6	514.33	0.1498 ± 0.0200	11.5231
*ZmC3H10* & *ZmC3H34*	3	275.50	0.2019 ± 0.0367	15.5308
*ZmC3H10* & *ZmC3H43*	4	291.83	0.1990 ± 0.0326	15.3077
*ZmC3H34* & *ZmC3H43*	5	310.50	0.0157 ± 0.0352	1.2077
*OsC3H24* & *SbC3H2*	14	572.67	0.8338 ± 0.4394	64.1385
*OsC3H24* & *ZmC3H4*	4	571.75	0.6299 ± 0.2791	48.4539
*OsC3H24* & *ZmC3H28*	5	359.50	0.5979 ± 0.1667	45.9923
*SbC3H2* & *ZmC3H4*	4	570.75	0.1853 ± 0.1206	14.2538
*SbC3H2* & *ZmC3H28*	5	359.92	0.1921 ± 0.0503	14.7769
*ZmC3H4* & *ZmC3H28*	4	361.00	0.2170 ± 0.0754	16.6923
*OsC3H33* & *SbC3H45*	9	458.25	0.5963 ± 0.1419	45.8692
*SbC3H45* & *ZmC3H54*	3	269.67	0.2567 ± 0.0433	19.7462
