# Supplementary material for: Significant Microsynteny with New Evolutionary Highlights Is Detected through Comparative Genomic Sequence Analysis of Maize CCCH IX Gene Subfamily
Source: Int J Genomics. 2015 Oct 11;2015:824287. doi: 10.1155/2015/824287 (PMC4619961; doi:10.1155/2015/824287)
Supplement: Supplementary file 1 — For the big data, we put supplementary figures and tables in Supplementary Material. Supplementary Figure 1 showed expression profiles of CCCH IX genes across different tissues in maize; Supplementary Figure 2 showed phylogenetic relationship of CCCH IX genes constructed by NJ, ML, and MP methods; Supplementary Figure 3 showed sliding window analysis of duplicated CCCH IX genes in three grass species. Supplementary TABLE 3 listed CCCH genes in Sorghum bicolor. Circos use steps: give the detailed steps to draw figure 4 by circos-0.54 program. [file 824287.f1.zip › 824287.f1/figures, tables, and supplementary materials/Figure and Table Legends.docx]

**Figure Legends**.

**Figure 1** Chromosomal locations of CCCH IX genes in three Graminaceae species (*O. sativa*, *S. bicolor* and *Z. mays*). The 27 CCCH IX genes are randomly distributed on chromosomes, including nine rice genes, six sorghum genes, and twelve maize genes.

**Figure 2 a:** Phylogenetic tree of CCCH IX proteins from *O. sativa*, *S. bicolor*, *and Z. mays*. This tree was constructed using the MEGA 6.0 program by the N-J method with 1,000 bootstrap replicates based on amino acid sequence. The tree is divided into four clades (clades I- IV). **b:** Exon-intron structures of 27 CCCH IX genes in three species. Exons and introns are indicated by green thick lines and thin gray lines, respectively. The untranslated regions (UTRs) are indicated by blue lines.

**Figure 3** Alignment of the amino acid sequences of CCCH IX proteins in three grass species. Identical (100%), conservative (75- 99%), and blocks (50- 74%) of similar amino acid residues are shaded in deep blue, dark pink, and light blue, respectively. The conserved CCCH zinc finger motifs are indicated by straight lines.

**Figure 4** Extensive microsynteny of CCCH regions across *O. sativa*, *S. bicolor* and *Z. mays.* *O. sativa* chromosomes (labeled Os) are indicated by orange boxes. *S. bicolor* and *Z. mays* chromosomes (labeled Sb and Zm, respectively) are shown in blue and green, respectively. CCCH IX regions in the three grasses are shown in the circle. Black lines show syntenic relationships.

**Figure 5** Microsynteny maps of CCCH IX genes in grasses. Red arrows represent anchor (CCCH IX) genes, and upstream and downstream genes are represented by black arrows. All genes are numbered from left to right for each segment. Black lines connect conserved gene pairs.

**Figure 6 a:** Distribution of synonymous distance (Ks) between paralogous genes flanking duplicated CCCH IX genes in the three grass species. The histogram depicts the number of duplicate gene pairs (y-axis) versus synonymous distance between pairs (x-axis). CCCH IX blocks experienced whole genome duplication (WGD) in the first stage of their evolution and large-scale duplication (LSD) in the second stage. **b:**  Ka/Ks ratios of duplicated CCCH IX genes and their flanking paralogs in the three grasses. The y and x axes denote the Ka/Ks ratio and synonymous distance for each pair, respectively.

**Table 1** The 27 CCCH IX genes identified in three species and their sequence characteristics (gene ID, ORF, MW, PI, and chromosome locations).

**Table 2** Estimate the dates of large-scale duplication events in three grasses.

**Supporting Information Legends**

**Supplementary Figure 1** Expression profiles of CCCH IX genes across different tissues. The genome-wide microarray data were obtained from the NimbleGen microarray provided by Sekhon et al. [[12](#_ENREF_12)].

**Supplementary Figure 2** Phylogenetic relationship of CCCH IX genes constructed using MEGA6.0 by the neighbor-joining method, Maximum likelihood method, and Maximum parsimony method. a: NJ method parameters: Poisson correction, pairwise deletion, and bootstrapping (1,000 replicates); b: ML method parameters: bootstrap = 1,000 replicates, amino acid substitution model, Jones-Taylor-Thornton matrix; c: MP method parameters: bootstrap = 1,000 replicates.

**Supplementary Figure 3** Sliding window analysis of duplicated CCCH IX genes in three grass species. The window size is 150 bp; step size, 9 bp. The X-axis denotes the nucleotide position. The Y-axis denotes the Ka/Ks ratio. The gaps represent Ka/Ks ratios that could not be computed.

**Supplementary Table 1** List of 55 CCCH genes identified in *Sorghum bicolor*.
