# Supplementary material for: Significant Microsynteny with New Evolutionary Highlights Is Detected through Comparative Genomic Sequence Analysis of Maize CCCH IX Gene Subfamily
Source: Int J Genomics. 2015 Oct 11;2015:824287. doi: 10.1155/2015/824287 (PMC4619961; doi:10.1155/2015/824287)
Supplement: Supplementary file 1 — For the big data, we put supplementary figures and tables in Supplementary Material. Supplementary Figure 1 showed expression profiles of CCCH IX genes across different tissues in maize; Supplementary Figure 2 showed phylogenetic relationship of CCCH IX genes constructed by NJ, ML, and MP methods; Supplementary Figure 3 showed sliding window analysis of duplicated CCCH IX genes in three grass species. Supplementary TABLE 3 listed CCCH genes in Sorghum bicolor. Circos use steps: give the detailed steps to draw figure 4 by circos-0.54 program. [file 824287.f1.zip › 824287.f1/figures, tables, and supplementary materials/Figures and Tables/Tables/supplementary table1.docx]

Supplementary Table 1 List of 55 CCCH genes identified in *Sorghum bicolor*.

| Identifier | Name | Chromosome | Start | End |
| --- | --- | --- | --- | --- |
| SB01G002770 | SbC3H1 | 1 | 2280279 | 2282517 |
| SB01G011150 | SbC3H2 | 1 | 10009063 | 10012269 |
| SB01G011290 | SbC3H3 | 1 | 10144382 | 10145397 |
| SB01G011590 | SbC3H4 | 1 | 10409990 | 10416003 |
| SB01G017290 | SbC3H5 | 1 | 17783795 | 17794688 |
| SB01G036490 | SbC3H6 | 1 | 60090967 | 60093996 |
| SB01G037830 | SbC3H7 | 1 | 61393524 | 61395495 |
| SB01G044515 | SbC3H8 | 1 | 67631156 | 67632142 |
| SB02G023040 | SbC3H9 | 2 | 56346506 | 56350763 |
| SB02G036710 | SbC3H10 | 2 | 71102658 | 71104964 |
| SB02G043180 | SbC3H11 | 2 | 76937595 | 76940453 |
| SB03G003110 | SbC3H12 | 3 | 3207828 | 3209693 |
| SB03G004260 | SbC3H13 | 3 | 4571541 | 4572453 |
| SB03G009590 | SbC3H14 | 3 | 10339021 | 10341737 |
| SB03G009880 | SbC3H15 | 3 | 10635475 | 10643421 |
| SB03G009920 | SbC3H16 | 3 | 10692896 | 10698230 |
| SB03G009930 | SbC3H17 | 3 | 10703836 | 10709394 |
| SB03G025780 | SbC3H18 | 3 | 51832516 | 51836486 |
| SB03G030670 | SbC3H19 | 3 | 58894441 | 58896902 |
| SB03G039020 | SbC3H20 | 3 | 66781261 | 66786165 |
| SB04G004243 | SbC3H21 | 4 | 4039138 | 4039395 |
| SB04G006450 | SbC3H22 | 4 | 6459118 | 6461711 |
| SB04G009270 | SbC3H23 | 4 | 11394707 | 11399775 |
| SB04G022810 | SbC3H24 | 4 | 52406782 | 52408770 |
| SB04G022813 | SbC3H25 | 4 | 52410642 | 52412026 |
| SB04G029760 | SbC3H26 | 4 | 59816696 | 59820504 |
| SB04G030200 | SbC3H27 | 4 | 60223218 | 60227032 |
| SB04G031930 | SbC3H28 | 4 | 61826609 | 61832603 |
| SB04G035680 | SbC3H29 | 4 | 65574462 | 65578469 |
| SB04G038370 | SbC3H30 | 4 | 67805275 | 67807992 |
| SB05G001540 | SbC3H31 | 5 | 1610255 | 1614884 |
| SB05G013190 | SbC3H32 | 5 | 27354787 | 27382604 |
| SB06G000450 | SbC3H33 | 6 | 468828 | 476319 |
| SB06G001050 | SbC3H34 | 6 | 1504354 | 1511180 |
| SB06G014350 | SbC3H35 | 6 | 39704438 | 39710851 |
| SB06G031850 | SbC3H36 | 6 | 60164114 | 60170159 |
| SB06G032000 | SbC3H37 | 6 | 60271362 | 60274541 |
| SB06G032480 | SbC3H38 | 6 | 60611879 | 60615409 |
| SB07G002750 | SbC3H39 | 7 | 2924823 | 2932897 |
| SB07G004075 | SbC3H40 | 7 | 5128794 | 5133759 |
| SB07G004080 | SbC3H41 | 7 | 5133816 | 5134529 |
| SB08G001980 | SbC3H42 | 8 | 2010176 | 2013391 |
| SB08G012360 | SbC3H43 | 8 | 32307877 | 32329902 |
| SB08G016640 | SbC3H44 | 8 | 44663680 | 44665480 |
| SB09G002390 | SbC3H45 | 9 | 2607622 | 2610024 |
| SB09G005300 | SbC3H46 | 9 | 6813175 | 6815685 |
| SB09G006050 | SbC3H47 | 9 | 8731871 | 8733975 |
| SB09G028530 | SbC3H48 | 9 | 57414613 | 57417199 |
| SB09G029330 | SbC3H49 | 9 | 58018089 | 58019608 |
| SB10G004840 | SbC3H50 | 10 | 4282860 | 4286546 |
| SB10G024960 | SbC3H51 | 10 | 54149365 | 54157246 |
| SB10G026940 | SbC3H52 | 10 | 56345471 | 56351057 |
| SB10G027200 | SbC3H53 | 10 | 56906869 | 56912844 |
| SB10G027530 | SbC3H54 | 10 | 57329867 | 57330286 |
| SB10G029250 | SbC3H55 | 10 | 59070250 | 59074396 |
