# Supplementary material for: Significant Microsynteny with New Evolutionary Highlights Is Detected through Comparative Genomic Sequence Analysis of Maize CCCH IX Gene Subfamily
Source: Int J Genomics. 2015 Oct 11;2015:824287. doi: 10.1155/2015/824287 (PMC4619961; doi:10.1155/2015/824287)
Supplement: Supplementary file 1 — For the big data, we put supplementary figures and tables in Supplementary Material. Supplementary Figure 1 showed expression profiles of CCCH IX genes across different tissues in maize; Supplementary Figure 2 showed phylogenetic relationship of CCCH IX genes constructed by NJ, ML, and MP methods; Supplementary Figure 3 showed sliding window analysis of duplicated CCCH IX genes in three grass species. Supplementary TABLE 3 listed CCCH genes in Sorghum bicolor. Circos use steps: give the detailed steps to draw figure 4 by circos-0.54 program. [file 824287.f1.zip › 824287.f1/figures, tables, and supplementary materials/Figures and Tables/Tables/table1.docx]

**Table 1** The 27 CCCH IX genes identified in three species and their sequence characteristics (gene ID, ORF, MW, PI, and chromosome locations).

| Gene name | Gene identifer | ORF (aa) | MW (Da) | pI | Chromosome | chromosomal localization  Star End | |
| --- | --- | --- | --- | --- | --- | --- | --- |
| OsC3H2 | LOC_Os01g09620.1 | 386 | 41422.51 | 6.41 | Os1 | 4949047 | 4951126 |
| OsC3H10 | LOC_Os01g53650.1 | 225 | 24982.17 | 6.06 | Os1 | 30824689 | 30825670 |
| OsC3H24 | LOC_Os03g49170.1 | 764 | 81043.54 | 6.63 | Os3 | 28008600 | 28012204 |
| OsC3H33 | LOC_Os05g03760.1 | 601 | 63235.89 | 8.65 | Os5 | 1662021 | 1664438 |
| OsC3H35 | LOC_Os05g10670.1 | 464 | 49684.7 | 9.02 | Os5 | 5846045 | 5848291 |
| OsC3H37 | LOC_Os05g45020.1 | 255 | 28257.88 | 5.39 | Os5 | 26171092 | 26172349 |
| OsC3H50 | LOC_Os07g38090.1 | 657 | 69391.93 | 6.61 | Os7 | 22840986 | 22843954 |
| OsC3H52 | LOC_Os07g47240.1 | 280 | 31590.77 | 8.04 | Os7 | 28233256 | 28234642 |
| OsC3H67 | LOC_Os12g33090.1 | 619 | 64725.96 | 6.04 | Os12 | 20018555 | 20021302 |
| ZmC3H4 | GRMZM2G180979_P01 | 746 | 79771.77 | 6.4 | Zm1 | 263731420 | 263734946 |
| ZmC3H10 | GRMZM2G099622_P03 | 360 | 37921.57 | 6.37 | Zm2 | 205904041 | 205906953 |
| ZmC3H12 | GRMZM5G853245_P03 | 370 | 39786.59 | 6.46 | Zm3 | 6681798 | 6683631 |
| ZmC3H28 | GRMZM5G845366_P01 | 482 | 52092.46 | 7.97 | Zm5 | 12382780 | 12384893 |
| ZmC3H34 | AC233871.1_FGP008 | 416 | 45437.96 | 8.78 | Zm6 | 1823029 | 1825102 |
| ZmC3H38 | GRMZM5G801627_P01 | 394 | 42104.18 | 8.02 | Zm6 | 132663265 | 132665063 |
| ZmC3H39 | GRMZM2G004795_P01 | 270 | 29691.44 | 5.67 | Zm6 | 160013893 | 160016282 |
| ZmC3H43 | GRMZM5G842019_P01 | 656 | 69821.35 | 6.88 | Zm7 | 158677416 | 158680576 |
| ZmC3H51 | GRMZM2G173124_P03 | 378 | 40178.89 | 6.46 | Zm8 | 20669510 | 20671733 |
| ZmC3H53 | GRMZM2G093404_P01 | 262 | 28025.75 | 6.4 | Zm8 | 124841672 | 124843159 |
| ZmC3H54 | GRMZM2G117007_P01 | 372 | 40034.27 | 8.65 | Zm8 | 131041707 | 131043172 |
| ZmC3H63 | GRMZM2G027298_P01 | 594 | 62657.48 | 5.71 | Zm10 | 27949011 | 27951381 |
| SbC3H2 | SB01G011150 | 745 | 79472.39 | 6.51 | Sb1 | 10009063 | 10012269 |
| SbC3H10 | SB02G036710 | 680 | 72441.2 | 6.88 | Sb2 | 71102658 | 71104964 |
| SbC3H12 | SB03G003110 | 350 | 37662.05 | 8.2 | Sb3 | 3207828 | 3209693 |
| SbC3H44 | SB08G016640 | 533 | 56473.43 | 5.89 | Sb8 | 44663680 | 44665480 |
| SbC3H45 | SB09G002390 | 611 | 64185.57 | 8.21 | Sb9 | 2607622 | 2610024 |
| SbC3H47 | SB09G006050 | 399 | 42664.56 | 6.21 | Sb9 | 8731871 | 8733975 |

Open reading frame (ORF), molecular weight (MW), isoelectric point (IP)
