# Supplementary material for: Significant Microsynteny with New Evolutionary Highlights Is Detected through Comparative Genomic Sequence Analysis of Maize CCCH IX Gene Subfamily
Source: Int J Genomics. 2015 Oct 11;2015:824287. doi: 10.1155/2015/824287 (PMC4619961; doi:10.1155/2015/824287)
Supplement: Supplementary file 1 — For the big data, we put supplementary figures and tables in Supplementary Material. Supplementary Figure 1 showed expression profiles of CCCH IX genes across different tissues in maize; Supplementary Figure 2 showed phylogenetic relationship of CCCH IX genes constructed by NJ, ML, and MP methods; Supplementary Figure 3 showed sliding window analysis of duplicated CCCH IX genes in three grass species. Supplementary TABLE 3 listed CCCH genes in Sorghum bicolor. Circos use steps: give the detailed steps to draw figure 4 by circos-0.54 program. [file 824287.f1.zip › 824287.f1/figures, tables, and supplementary materials/Supplementary Materials.docx]

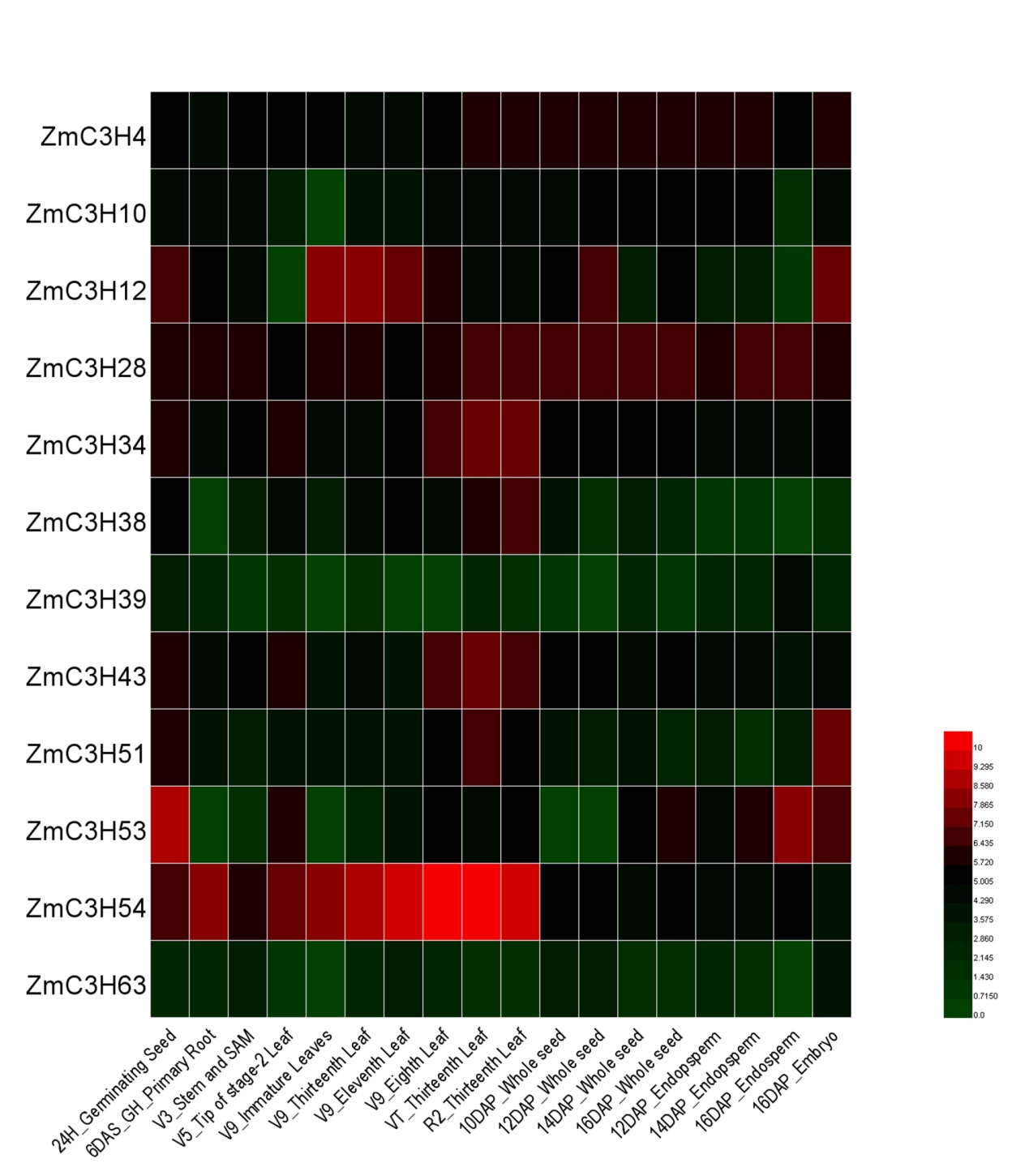


**Supplementary Figure 1:** Expression profiles of CCCH IX genes across different tissues. The genome-wide microarray data were obtained from the NimbleGen microarray provided by Sekhon et al. [[12](#_ENREF_12)].


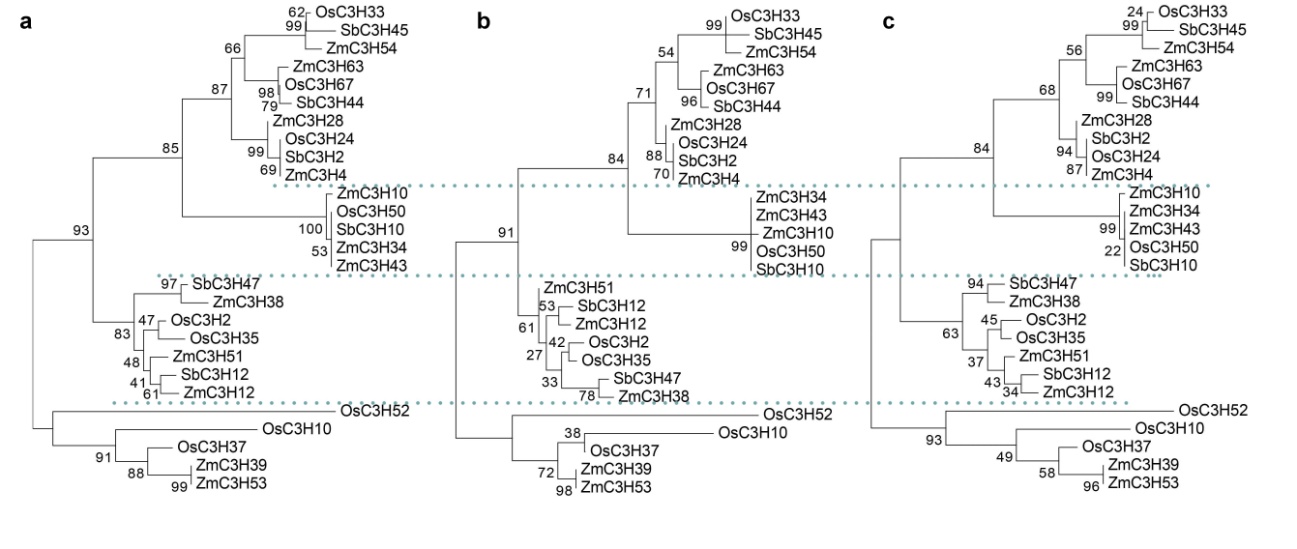


**Supplementary Figure 2:** Phylogenetic relationship of CCCH IX genes constructed using MEGA6.0 by the neighbor-joining method, Maximum likelihood method, and Maximum parsimony method. a: NJ method parameters: Poisson correction, pairwise deletion, and bootstrapping (1,000 replicates); b: ML method parameters: bootstrap = 1,000 replicates, amino acid substitution model, Jones-Taylor-Thornton matrix; c: MP method parameters: bootstrap = 1,000 replicates.


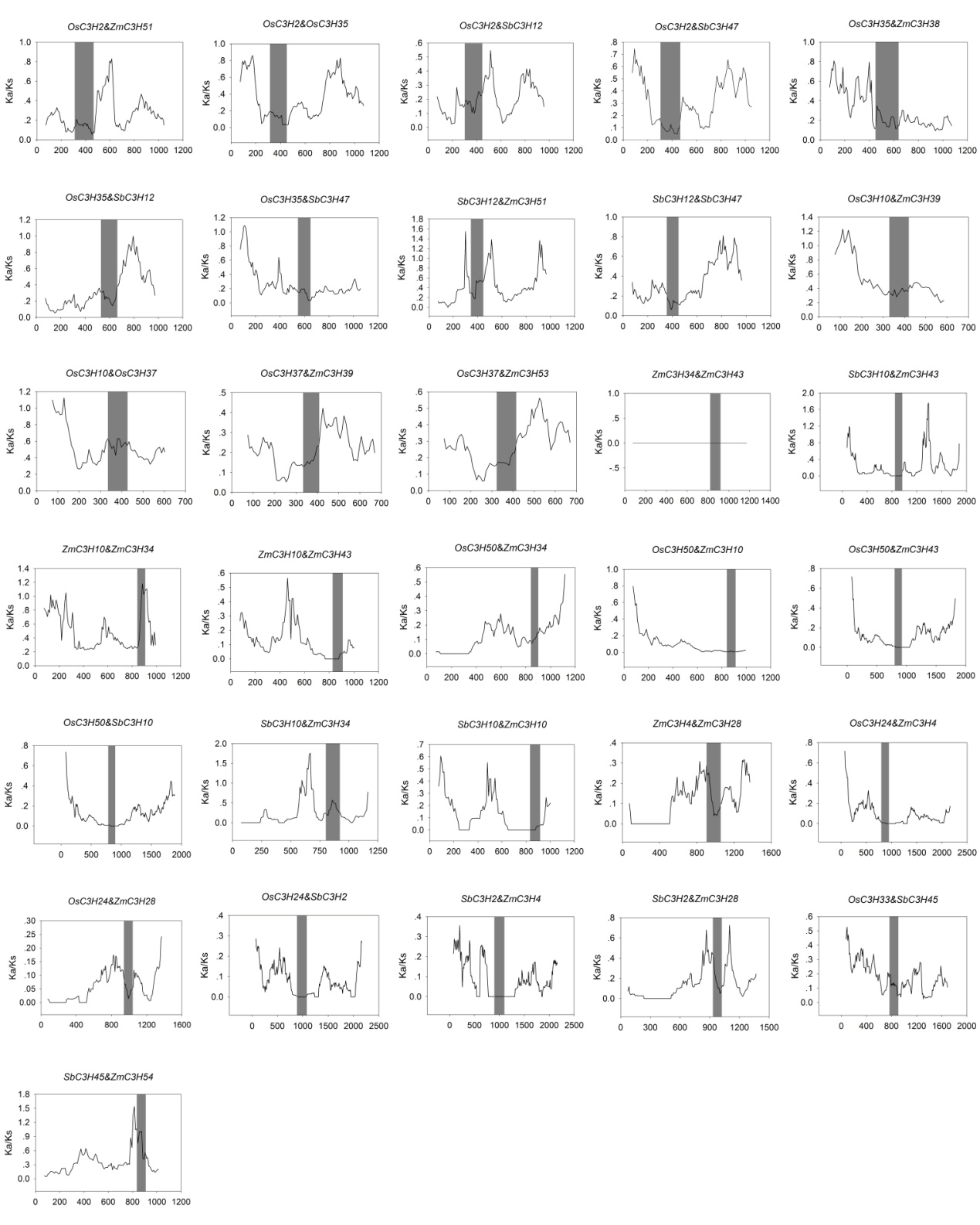


**Supplementary Figure 3:** Sliding window analysis of duplicated CCCH IX genes in three grass species. The window size is 150 bp; step size, 9 bp. The X-axis denotes the nucleotide position. The Y-axis denotes the Ka/Ks ratio. The gaps represent Ka/Ks ratios that could not be computed.

TABLE S1: List of 55 CCCH genes identified in *Sorghum bicolor*.

| Identifier | Name | Chromosome | Start | End |
| --- | --- | --- | --- | --- |
| SB01G002770 | SbC3H1 | 1 | 2280279 | 2282517 |
| SB01G011150 | SbC3H2 | 1 | 10009063 | 10012269 |
| SB01G011290 | SbC3H3 | 1 | 10144382 | 10145397 |
| SB01G011590 | SbC3H4 | 1 | 10409990 | 10416003 |
| SB01G017290 | SbC3H5 | 1 | 17783795 | 17794688 |
| SB01G036490 | SbC3H6 | 1 | 60090967 | 60093996 |
| SB01G037830 | SbC3H7 | 1 | 61393524 | 61395495 |
| SB01G044515 | SbC3H8 | 1 | 67631156 | 67632142 |
| SB02G023040 | SbC3H9 | 2 | 56346506 | 56350763 |
| SB02G036710 | SbC3H10 | 2 | 71102658 | 71104964 |
| SB02G043180 | SbC3H11 | 2 | 76937595 | 76940453 |
| SB03G003110 | SbC3H12 | 3 | 3207828 | 3209693 |
| SB03G004260 | SbC3H13 | 3 | 4571541 | 4572453 |
| SB03G009590 | SbC3H14 | 3 | 10339021 | 10341737 |
| SB03G009880 | SbC3H15 | 3 | 10635475 | 10643421 |
| SB03G009920 | SbC3H16 | 3 | 10692896 | 10698230 |
| SB03G009930 | SbC3H17 | 3 | 10703836 | 10709394 |
| SB03G025780 | SbC3H18 | 3 | 51832516 | 51836486 |
| SB03G030670 | SbC3H19 | 3 | 58894441 | 58896902 |
| SB03G039020 | SbC3H20 | 3 | 66781261 | 66786165 |
| SB04G004243 | SbC3H21 | 4 | 4039138 | 4039395 |
| SB04G006450 | SbC3H22 | 4 | 6459118 | 6461711 |
| SB04G009270 | SbC3H23 | 4 | 11394707 | 11399775 |
| SB04G022810 | SbC3H24 | 4 | 52406782 | 52408770 |
| SB04G022813 | SbC3H25 | 4 | 52410642 | 52412026 |
| SB04G029760 | SbC3H26 | 4 | 59816696 | 59820504 |
| SB04G030200 | SbC3H27 | 4 | 60223218 | 60227032 |
| SB04G031930 | SbC3H28 | 4 | 61826609 | 61832603 |
| SB04G035680 | SbC3H29 | 4 | 65574462 | 65578469 |
| SB04G038370 | SbC3H30 | 4 | 67805275 | 67807992 |
| SB05G001540 | SbC3H31 | 5 | 1610255 | 1614884 |
| SB05G013190 | SbC3H32 | 5 | 27354787 | 27382604 |
| SB06G000450 | SbC3H33 | 6 | 468828 | 476319 |
| SB06G001050 | SbC3H34 | 6 | 1504354 | 1511180 |
| SB06G014350 | SbC3H35 | 6 | 39704438 | 39710851 |
| SB06G031850 | SbC3H36 | 6 | 60164114 | 60170159 |
| SB06G032000 | SbC3H37 | 6 | 60271362 | 60274541 |
| SB06G032480 | SbC3H38 | 6 | 60611879 | 60615409 |
| SB07G002750 | SbC3H39 | 7 | 2924823 | 2932897 |
| SB07G004075 | SbC3H40 | 7 | 5128794 | 5133759 |
| SB07G004080 | SbC3H41 | 7 | 5133816 | 5134529 |
| SB08G001980 | SbC3H42 | 8 | 2010176 | 2013391 |
| SB08G012360 | SbC3H43 | 8 | 32307877 | 32329902 |
| SB08G016640 | SbC3H44 | 8 | 44663680 | 44665480 |
| SB09G002390 | SbC3H45 | 9 | 2607622 | 2610024 |
| SB09G005300 | SbC3H46 | 9 | 6813175 | 6815685 |
| SB09G006050 | SbC3H47 | 9 | 8731871 | 8733975 |
| SB09G028530 | SbC3H48 | 9 | 57414613 | 57417199 |
| SB09G029330 | SbC3H49 | 9 | 58018089 | 58019608 |
| SB10G004840 | SbC3H50 | 10 | 4282860 | 4286546 |
| SB10G024960 | SbC3H51 | 10 | 54149365 | 54157246 |
| SB10G026940 | SbC3H52 | 10 | 56345471 | 56351057 |
| SB10G027200 | SbC3H53 | 10 | 56906869 | 56912844 |
| SB10G027530 | SbC3H54 | 10 | 57329867 | 57330286 |
| SB10G029250 | SbC3H55 | 10 | 59070250 | 59074396 |

**Circos use steps**

1. downloaded Perl program installers and installing (<http://strawberryperl.com/>)
2. install Perl needful modules
3. download and install circos-0.54(<http://circos.ca/software/download/circos/>)
4. unzip “circos” file and make new folder “tmp” as output file
5. five configuration files
   1. “ideogram.conf” file, goes as follows

<ideogram>

<spacing>

default = 0.005r

break = 5u

</spacing>

#position configuration

radius = os1:0.5r;0s3:0.55r;0s5:0.6r;0s12:0.65r;zm1:0.65r;zm2:0.70r;zm3:0.75r;zm5:0.75r;zm6:0.80r;zm7:0.85r;zm8:0.90r;zm10:0.95r;sb1:1.05r;sb2:1.10r;sb3:1.15r;sb8:1.20r;sb9:1.25r

thickness = 100p

fill = yes

fill_color = black

stroke_thickness = 2

stroke_color = black

#label configuration

show_label = yes

label_font = bold

label_radius = dims(ideogram,radius) + 0.07r

label_with_tag = yes

label_size = 36

label_parallel = yes

#band configuration

show_bands = no

fill_bands = yes

band_stroke_thickness = 2

band_stroke_color = white

band_transparency = 4

</ideogram>

- 1. “ticks.conf” file, goes as follows

show_ticks = yes

show_tick_labels = yes

<ticks>

skip_first_label = no

skip_last_label = no

radius = dims(ideogram,radius_outer)

tick_separation = 3p

label_separation = 1p

multiplier = 1e-6

color = black

thickness = 4p

size = 20p

<tick>

spacing = 1u

show_label = no

thickness = 2p

color = dgrey

</tick>

<tick>

spacing = 5u

show_label = no

thickness = 3p

color = vdgrey

</tick>

<tick>

spacing = 10u

show_label = yes

label_size = 20p

label_offset = 10p

format = %d

grid = yes

grid_color = dgrey

grid_thickness = 1p

grid_start = 0.5r

grid_end = 0.999r

</tick>

</ticks>

- 1. “karyotype.pt.txt” file,goes as follows

chr - zm1 zm1 0 316040806 chr1

chr - zm2 zm2 0 248617369 chr2

chr - zm3 zm3 0 248617369 chr3

chr - zm4 zm4 0 253231104 chr4

chr - zm5 zm5 0 228484710 chr5

chr - zm6 zm6 0 177419059 chr6

chr - zm7 zm7 0 185388236 chr7

chr - zm8 zm8 0 184339660 chr8

chr - zm9 zm9 0 164416716 chr9

chr - zm10 zm10 0 157496115 chr10

chr - os1 Os1 0 45319455 chr1

chr -os2 Os2 0 37675336 chr2

chr -os3 Os3 0 38136709 chr3

chr -os5 Os5 0 31415337 chr4

chr -os7 Os7 0 31100764 chr5

chr -os8 Os8 0 29831987 chr6

chr -os9 Os9 0 24096276 chr7

chr -os10 Os10 0 24368906 chr8

chr -os11 Os11 0 30429676 chr9

chr -os12 Os12 0 28867297 chr10

chr - sb1 sb1 0 77594624 chr1

chr - sb2 sb2 0 81788928 chr2

chr - sb3 sb3 0 78643200 chr3

chr - sb4 sb4 0 71271710 chr4

chr - sb5 sb5 0 65536000 chr5

chr - sb6 sb6 0 28867297 chr6

chr - sb7 sb7 0 67381493 chr7

chr - sb8 sb8 0 58049167 chr8

chr - sb9 sb9 0 62264442 chr9

chr - sb10 sb10 0 63973621 chr10

- 1. “segdup.txt” file, goes as follows

segdup00001 os1 4949047 4951126 color=black

segdup00001 os5 5846045 5848291 color=black

segdup00002 os1 4949047 4951126 color=black

segdup00002 sb9 8731871 8733975 color=black

segdup00003 os1 4949047 4951126 color=black

segdup00003 zm820669510 20671733 color=black

segdup00004 os5 5846045 5848291 color=black

segdup00004 sb9 8731871 8733975 color=black

segdup00005 sb3 3207828 3209693 color=black

segdup00005 zm820669510 20671733 color=black

segdup00006 os1 4949047 4951126 color=black

segdup00006 sb3 3207828 3209693 color=black

segdup00007 os5 5846045 5848291 color=black

segdup00007 sb3 3207828 3209693 color=black

segdup00008 sb3 3207828 3209693 color=black

segdup00008 sb9 8731871 8733975 color=black

segdup00009 os5 5846045 5848291 color=black

segdup00009 zm6 132663265 132665063 color=black

segdup000010 os1 30824689 30825670 color=black

segdup000010 os5 26171092 26172349 color=black

segdup000011 os5 26171092 26172349 color=black

segdup000011 zm6 160013893 160016282 color=black

segdup000012 os1 30824689 30825670 color=black

segdup000012 zm6 160013893 160016282 color=black

segdup000013 os5 26171092 26172349 color=black

segdup000013 zm8 124841672 124843159 color=black

segdup000014 os7 22840986 22843954 color=black

segdup000014 os7 28233256 28234642 color=black

segdup000015 os7 22840986 22843954 color=black

segdup000015 sb2 71102658 71104964 color=black

segdup000016 sb2 71102658 71104964 color=black

segdup000016 zm2 205904041 205906953 color=black

segdup000017 sb2 71102658 71104964 color=black

segdup000017 zm7 158677416 158680576 color=black

segdup000018 zm2 205904041 205906953 color=black

segdup000018 zm7 158677416 158680576 color=black

segdup000019 os7 22840986 22843954 color=black

segdup000019 zm2 205904041 205906953 color=black

segdup000020 os7 22840986 22843954 color=black

segdup000020 zm6 1823029 1825102 color=black

segdup000021 os7 22840986 22843954 color=black

segdup000021 zm7 158677416 158680576 color=black

segdup000022 sb2 71102658 71104964 color=black

segdup000022 zm6 1823029 1825102 color=black

segdup000023 zm2 205904041 205906953 color=black

segdup000023 zm6 1823029 1825102 color=black

segdup000024 zm6 1823029 1825102 color=black

segdup000024 zm7 158677416 158680576 color=black

segdup000025 os3 28008600 28012204 color=black

segdup000025 sb1 10009063 10012269 color=black

segdup000026 sb1 10009063 10012269 color=black

segdup000026 zm1 263731420 263734946 color=black

segdup000027 zm1 263731420 263734946 color=black

segdup000027 zm5 12382780 12384893 color=black

segdup000028 os3 28008600 28012204 color=black

segdup000028 zm1 263731420 263734946 color=black

segdup000029 os3 28008600 28012204 color=black

segdup000029 zm5 12382780 12384893 color=black

segdup000030 sb1 10009063 10012269 color=black

segdup000030 zm5 12382780 12384893 color=black

segdup000031 os5 1662021 1664438 color=black

segdup000031 sb9 2607622 2610024 color=black

segdup000032 sb9 2607622 2610024 color=black

segdup000032 zm8 131041707 131043172 color=black

- 1. “circos.conf” file, goes as follows

<colors>

<<include etc/colors.conf>>

<<include etc/brewer.conf>>

</colors>

<fonts>

<<include etc/fonts.conf>>

</fonts>

<<include ideogram.conf>>

<<include ticks.conf>>

<image>

<<include etc/image.conf>>

</image>

karyotype = karyotype.pt.txt

chromosomes_units = 1000000

chromosomes = os1;os3;os5;os7;os12;zm1:220-310;zm2:150-240;zm5:0-90;zm6;zm7:100-180;zm8:0-160;sb1;sb2;sb3;sb8;sb9

# chromosomes_radius = hs2:0.9r;hs3:0.8r

# Links (bezier curves or straight lines) are defined in <links> blocks.

# Each link data set is defined within a named <link> block. The name

# of the block is arbitrary, but must be unique.

#

# As with highlights, parameters defined

# in the root of <links> affect all data sets and are considered

# global settings. Individual parameters value can be refined by

# values defined within <link> blocks, or additionally on each

# data line within the input file.

<links>

z = 0

radius = 0.975r

bezier_radius = 0.2r

<link segdup>

show = yes

color = black_a5

thickness = 10

file = segdup.txt

record_limit = 10000

</link>

</links>

<<include etc/housekeeping.conf>>

1. put the five files above below “circos” folder, and running command perl .\bin\circos -conf .\circos.conf
2. get output in “tmp” file
