# Supplementary figures and images for: Significant Microsynteny with New Evolutionary Highlights Is Detected through Comparative Genomic Sequence Analysis of Maize CCCH IX Gene Subfamily
Source: Int J Genomics. 2015 Oct 11;2015:824287. doi: 10.1155/2015/824287 (PMC4619961; doi:10.1155/2015/824287)

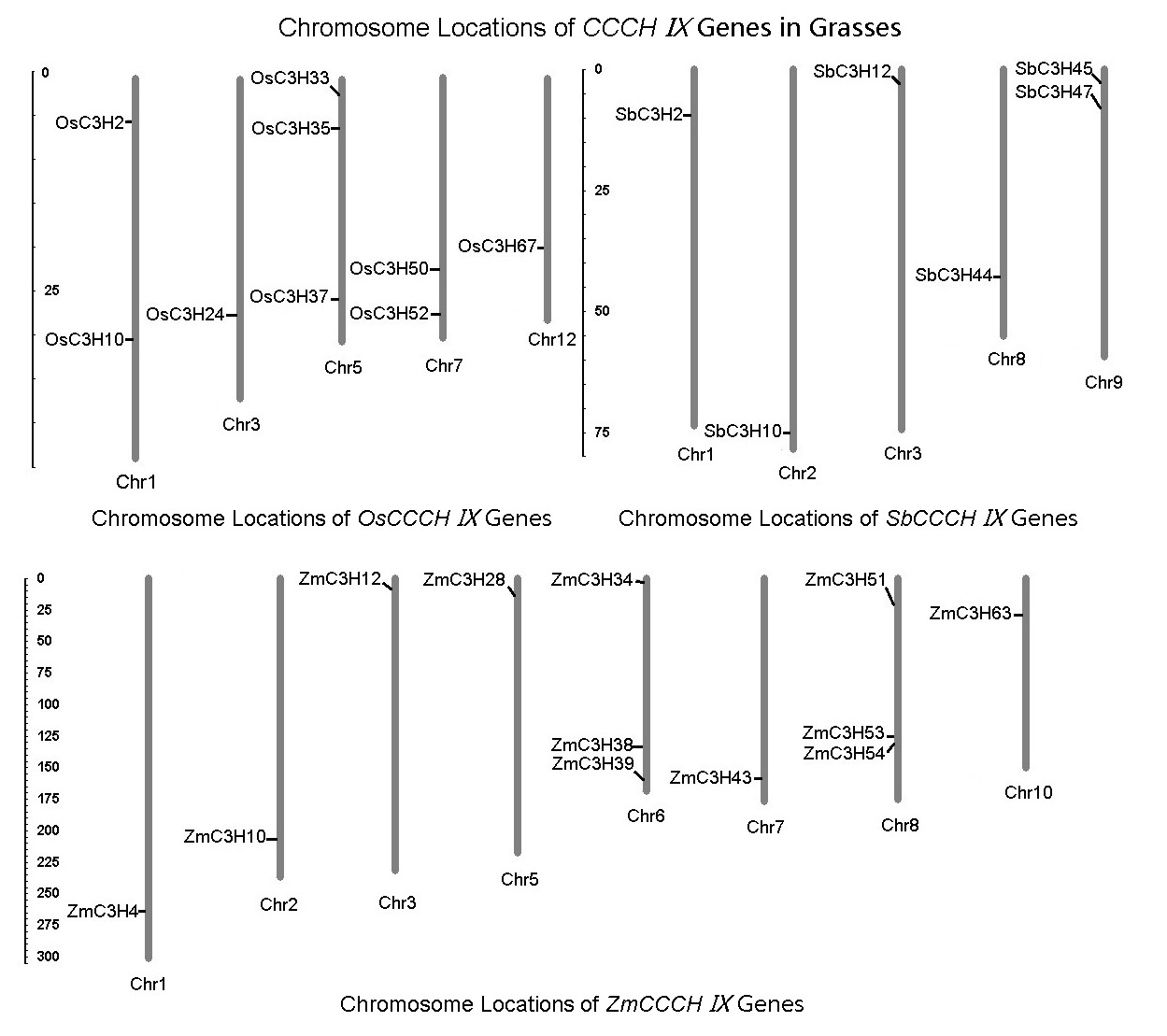

Supplement: Supplementary file 1 — For the big data, we put supplementary figures and tables in Supplementary Material. Supplementary Figure 1 showed expression profiles of CCCH IX genes across different tissues in maize; Supplementary Figure 2 showed phylogenetic relationship of CCCH IX genes constructed by NJ, ML, and MP methods; Supplementary Figure 3 showed sliding window analysis of duplicated CCCH IX genes in three grass species. Supplementary TABLE 3 listed CCCH genes in Sorghum bicolor. Circos use steps: give the detailed steps to draw figure 4 by circos-0.54 program. [file 824287.f1.zip › 824287.f1/figures, tables, and supplementary materials/Figures and Tables/Figures/figure 1.jpg]

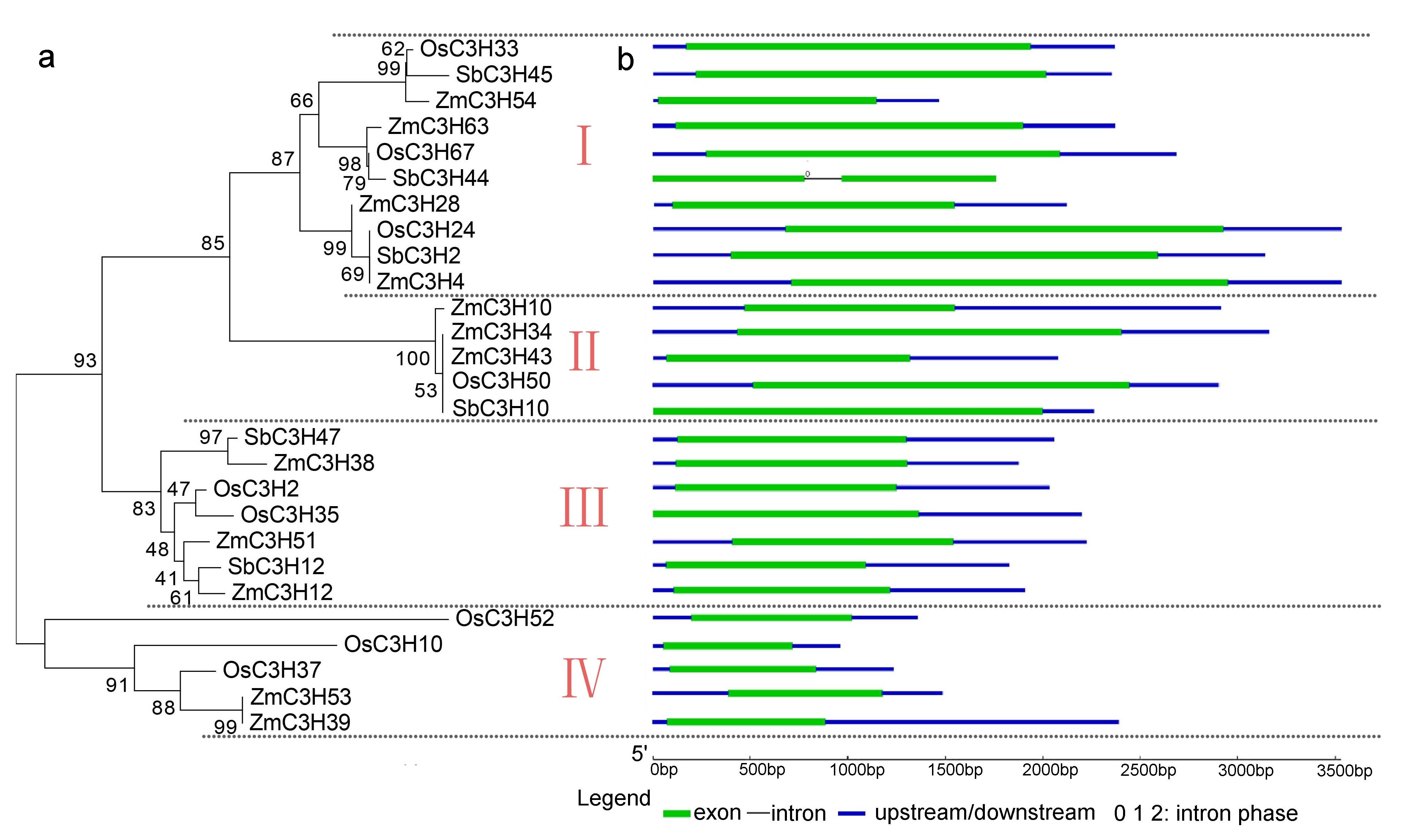

Supplement: Supplementary file 1 — For the big data, we put supplementary figures and tables in Supplementary Material. Supplementary Figure 1 showed expression profiles of CCCH IX genes across different tissues in maize; Supplementary Figure 2 showed phylogenetic relationship of CCCH IX genes constructed by NJ, ML, and MP methods; Supplementary Figure 3 showed sliding window analysis of duplicated CCCH IX genes in three grass species. Supplementary TABLE 3 listed CCCH genes in Sorghum bicolor. Circos use steps: give the detailed steps to draw figure 4 by circos-0.54 program. [file 824287.f1.zip › 824287.f1/figures, tables, and supplementary materials/Figures and Tables/Figures/figure 2.jpg]

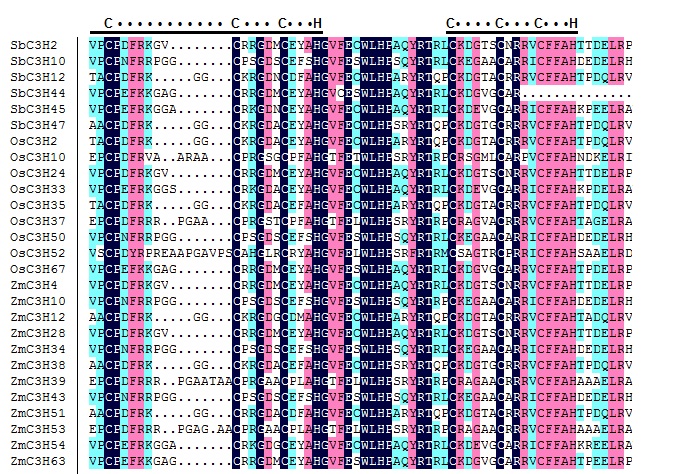

Supplement: Supplementary file 1 — For the big data, we put supplementary figures and tables in Supplementary Material. Supplementary Figure 1 showed expression profiles of CCCH IX genes across different tissues in maize; Supplementary Figure 2 showed phylogenetic relationship of CCCH IX genes constructed by NJ, ML, and MP methods; Supplementary Figure 3 showed sliding window analysis of duplicated CCCH IX genes in three grass species. Supplementary TABLE 3 listed CCCH genes in Sorghum bicolor. Circos use steps: give the detailed steps to draw figure 4 by circos-0.54 program. [file 824287.f1.zip › 824287.f1/figures, tables, and supplementary materials/Figures and Tables/Figures/figure 3.jpg]

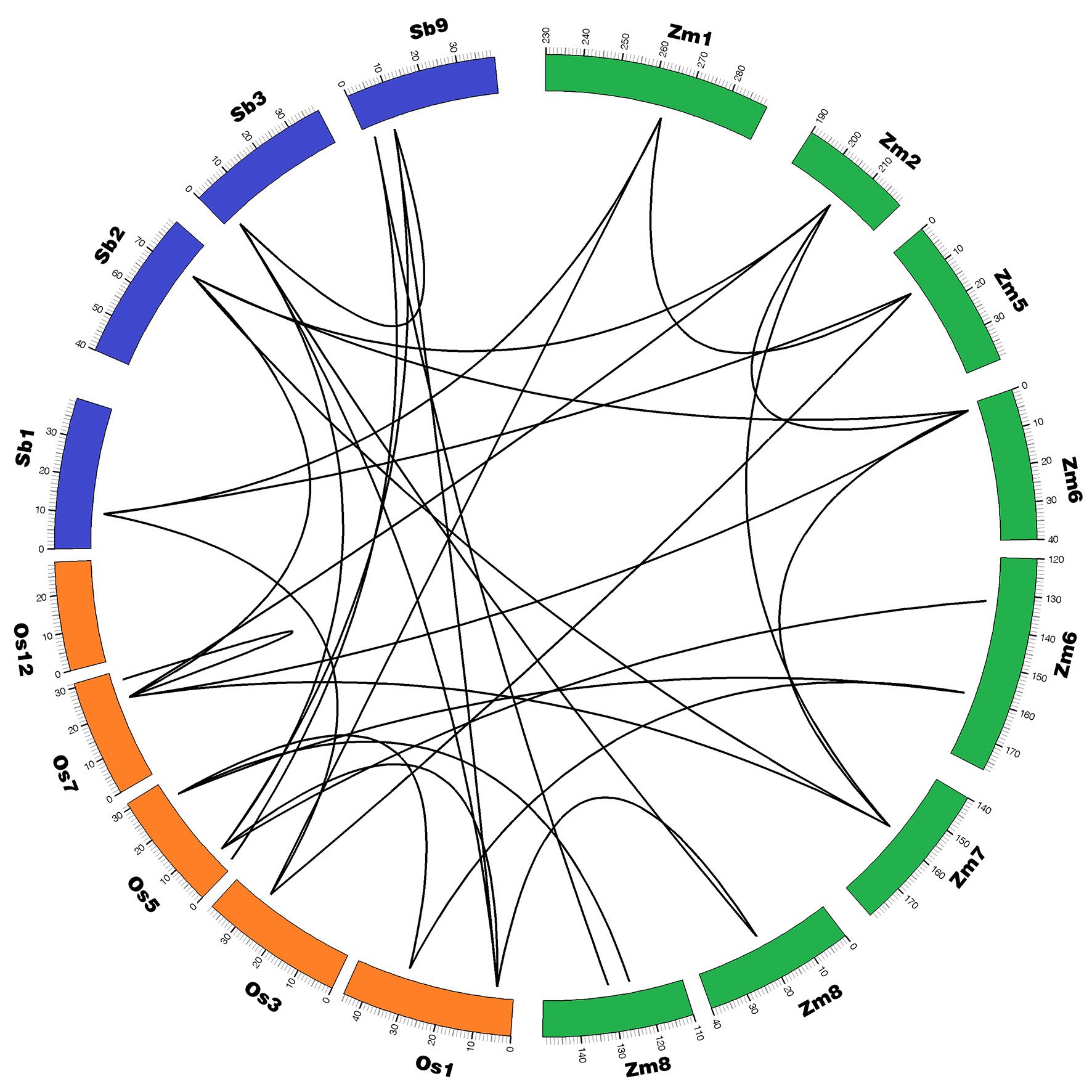

Supplement: Supplementary file 1 — For the big data, we put supplementary figures and tables in Supplementary Material. Supplementary Figure 1 showed expression profiles of CCCH IX genes across different tissues in maize; Supplementary Figure 2 showed phylogenetic relationship of CCCH IX genes constructed by NJ, ML, and MP methods; Supplementary Figure 3 showed sliding window analysis of duplicated CCCH IX genes in three grass species. Supplementary TABLE 3 listed CCCH genes in Sorghum bicolor. Circos use steps: give the detailed steps to draw figure 4 by circos-0.54 program. [file 824287.f1.zip › 824287.f1/figures, tables, and supplementary materials/Figures and Tables/Figures/figure4.jpg]

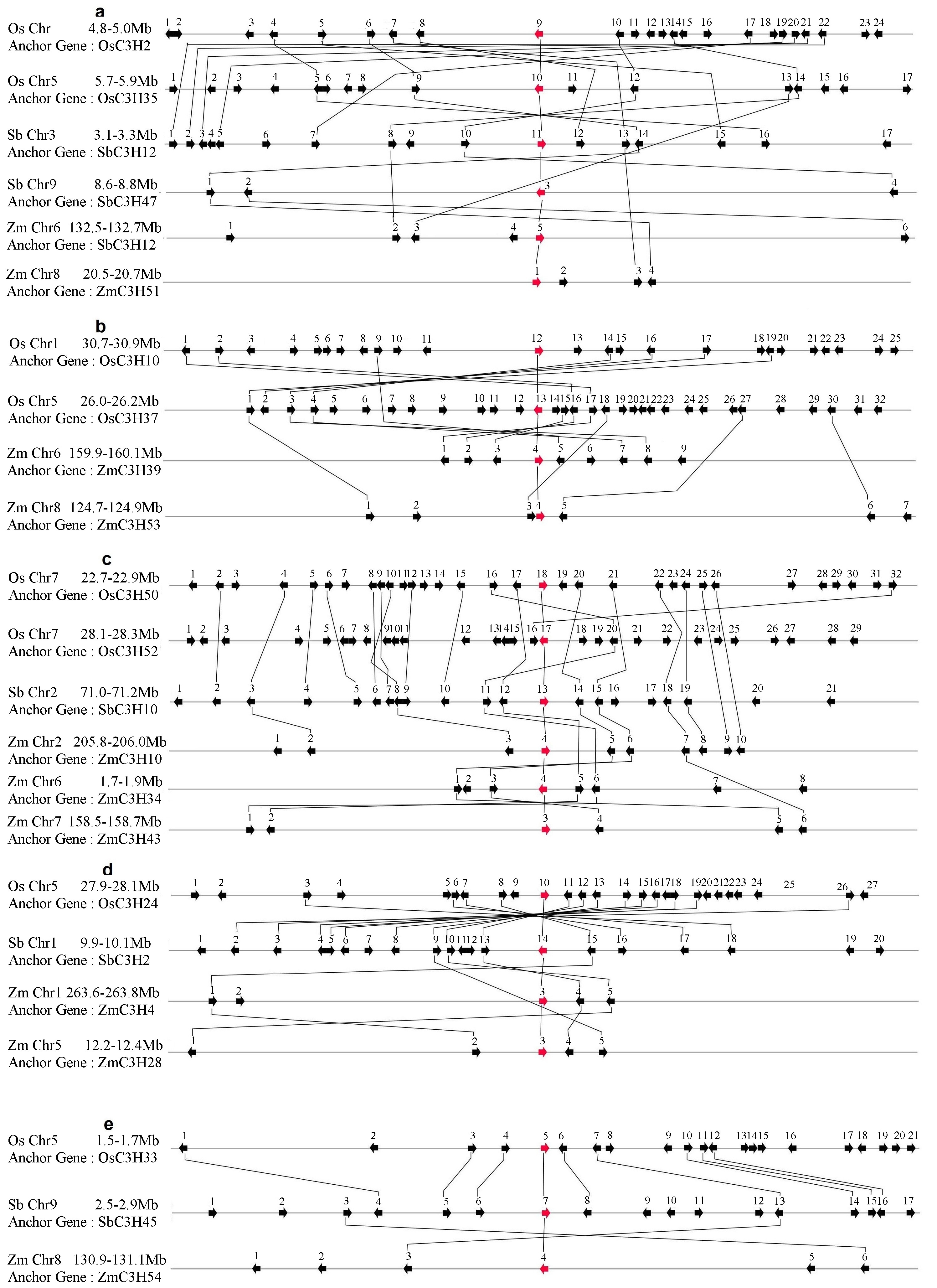

Supplement: Supplementary file 1 — For the big data, we put supplementary figures and tables in Supplementary Material. Supplementary Figure 1 showed expression profiles of CCCH IX genes across different tissues in maize; Supplementary Figure 2 showed phylogenetic relationship of CCCH IX genes constructed by NJ, ML, and MP methods; Supplementary Figure 3 showed sliding window analysis of duplicated CCCH IX genes in three grass species. Supplementary TABLE 3 listed CCCH genes in Sorghum bicolor. Circos use steps: give the detailed steps to draw figure 4 by circos-0.54 program. [file 824287.f1.zip › 824287.f1/figures, tables, and supplementary materials/Figures and Tables/Figures/figure5.jpg]

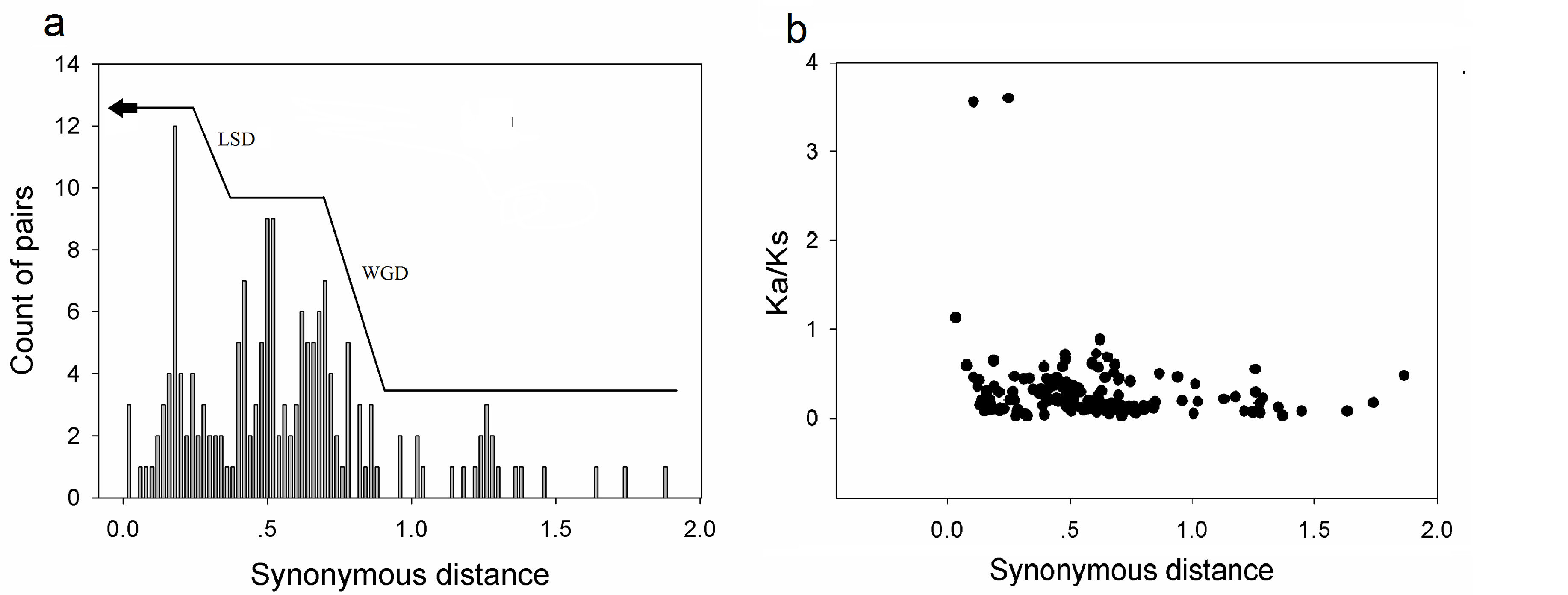

Supplement: Supplementary file 1 — For the big data, we put supplementary figures and tables in Supplementary Material. Supplementary Figure 1 showed expression profiles of CCCH IX genes across different tissues in maize; Supplementary Figure 2 showed phylogenetic relationship of CCCH IX genes constructed by NJ, ML, and MP methods; Supplementary Figure 3 showed sliding window analysis of duplicated CCCH IX genes in three grass species. Supplementary TABLE 3 listed CCCH genes in Sorghum bicolor. Circos use steps: give the detailed steps to draw figure 4 by circos-0.54 program. [file 824287.f1.zip › 824287.f1/figures, tables, and supplementary materials/Figures and Tables/Figures/figure6.jpg]

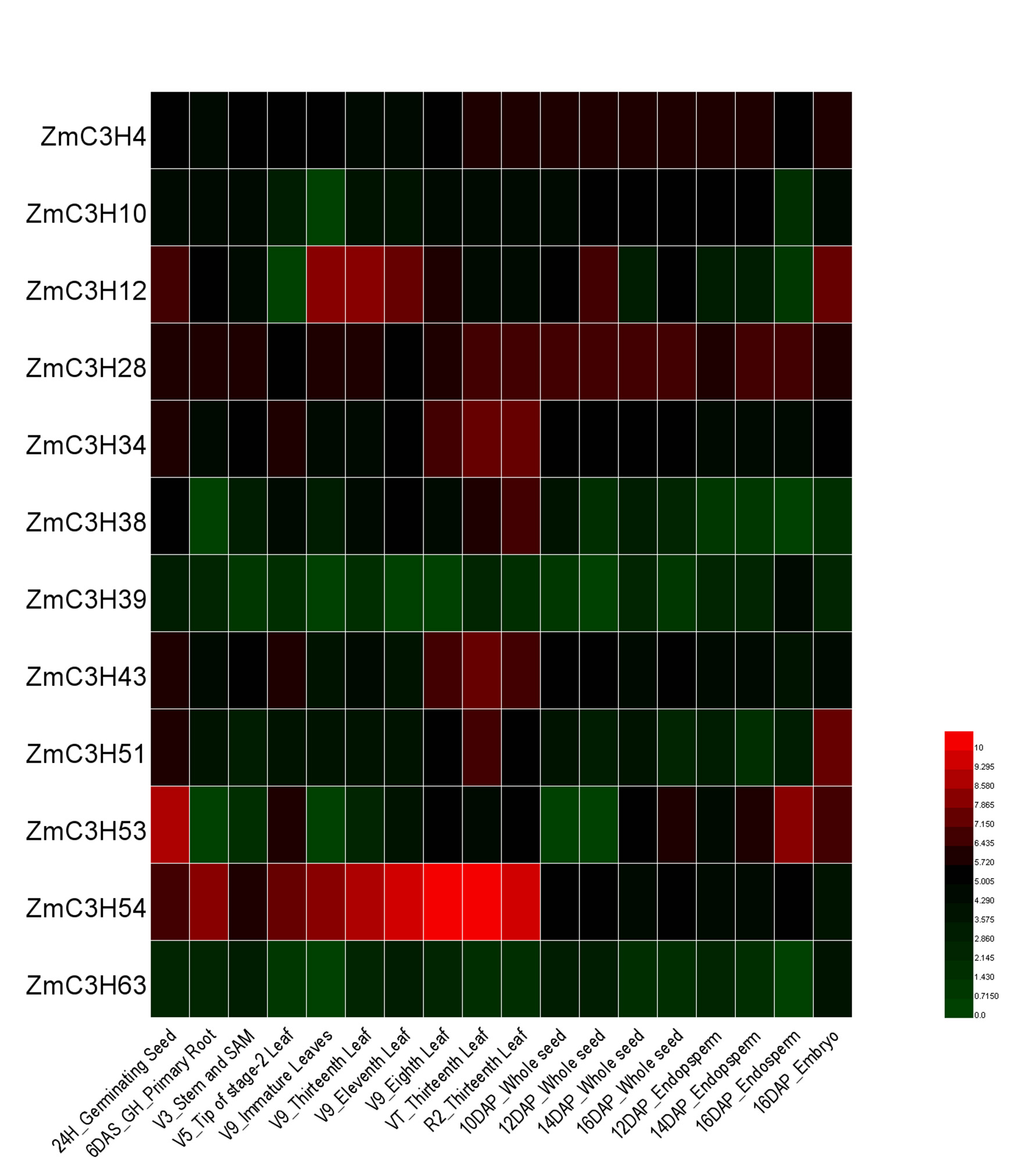

Supplement: Supplementary file 1 — For the big data, we put supplementary figures and tables in Supplementary Material. Supplementary Figure 1 showed expression profiles of CCCH IX genes across different tissues in maize; Supplementary Figure 2 showed phylogenetic relationship of CCCH IX genes constructed by NJ, ML, and MP methods; Supplementary Figure 3 showed sliding window analysis of duplicated CCCH IX genes in three grass species. Supplementary TABLE 3 listed CCCH genes in Sorghum bicolor. Circos use steps: give the detailed steps to draw figure 4 by circos-0.54 program. [file 824287.f1.zip › 824287.f1/figures, tables, and supplementary materials/Figures and Tables/Figures/supplementary figure1.jpg]

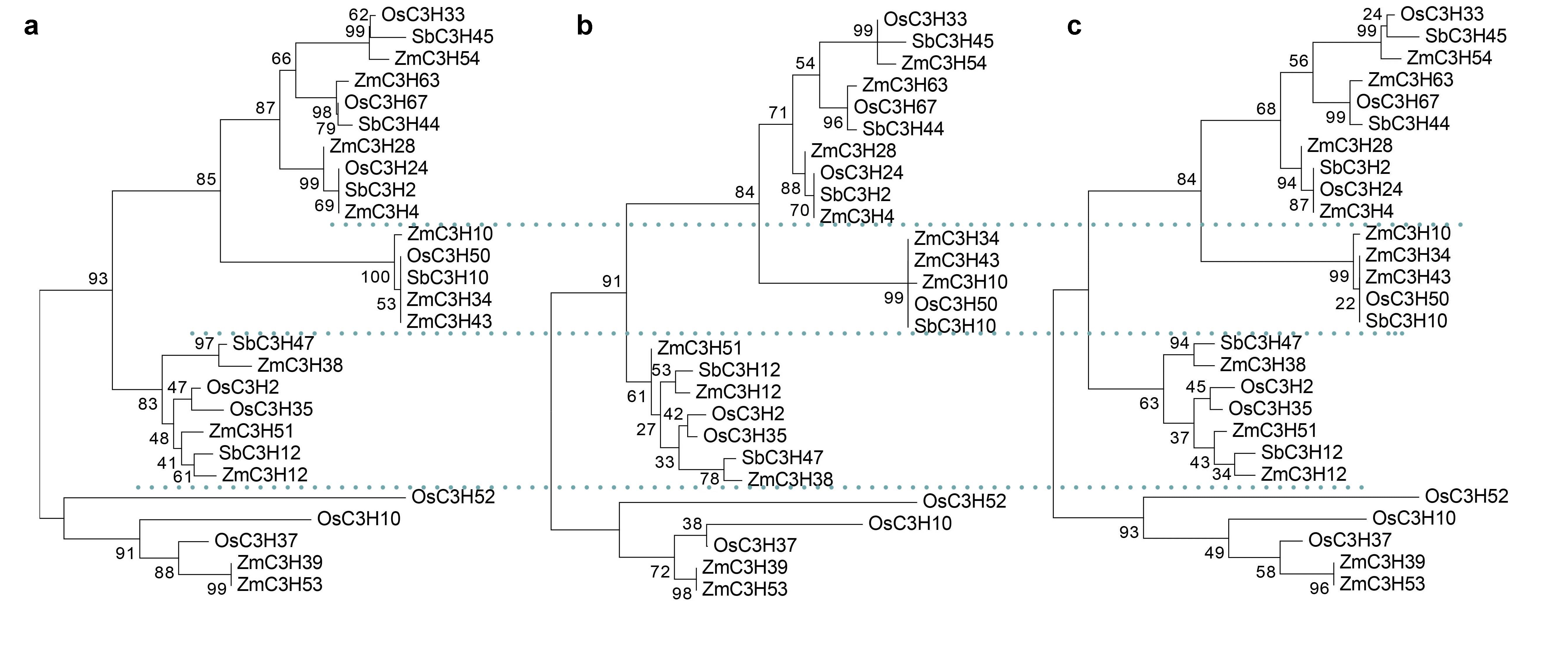

Supplement: Supplementary file 1 — For the big data, we put supplementary figures and tables in Supplementary Material. Supplementary Figure 1 showed expression profiles of CCCH IX genes across different tissues in maize; Supplementary Figure 2 showed phylogenetic relationship of CCCH IX genes constructed by NJ, ML, and MP methods; Supplementary Figure 3 showed sliding window analysis of duplicated CCCH IX genes in three grass species. Supplementary TABLE 3 listed CCCH genes in Sorghum bicolor. Circos use steps: give the detailed steps to draw figure 4 by circos-0.54 program. [file 824287.f1.zip › 824287.f1/figures, tables, and supplementary materials/Figures and Tables/Figures/supplementary figure2.jpg]

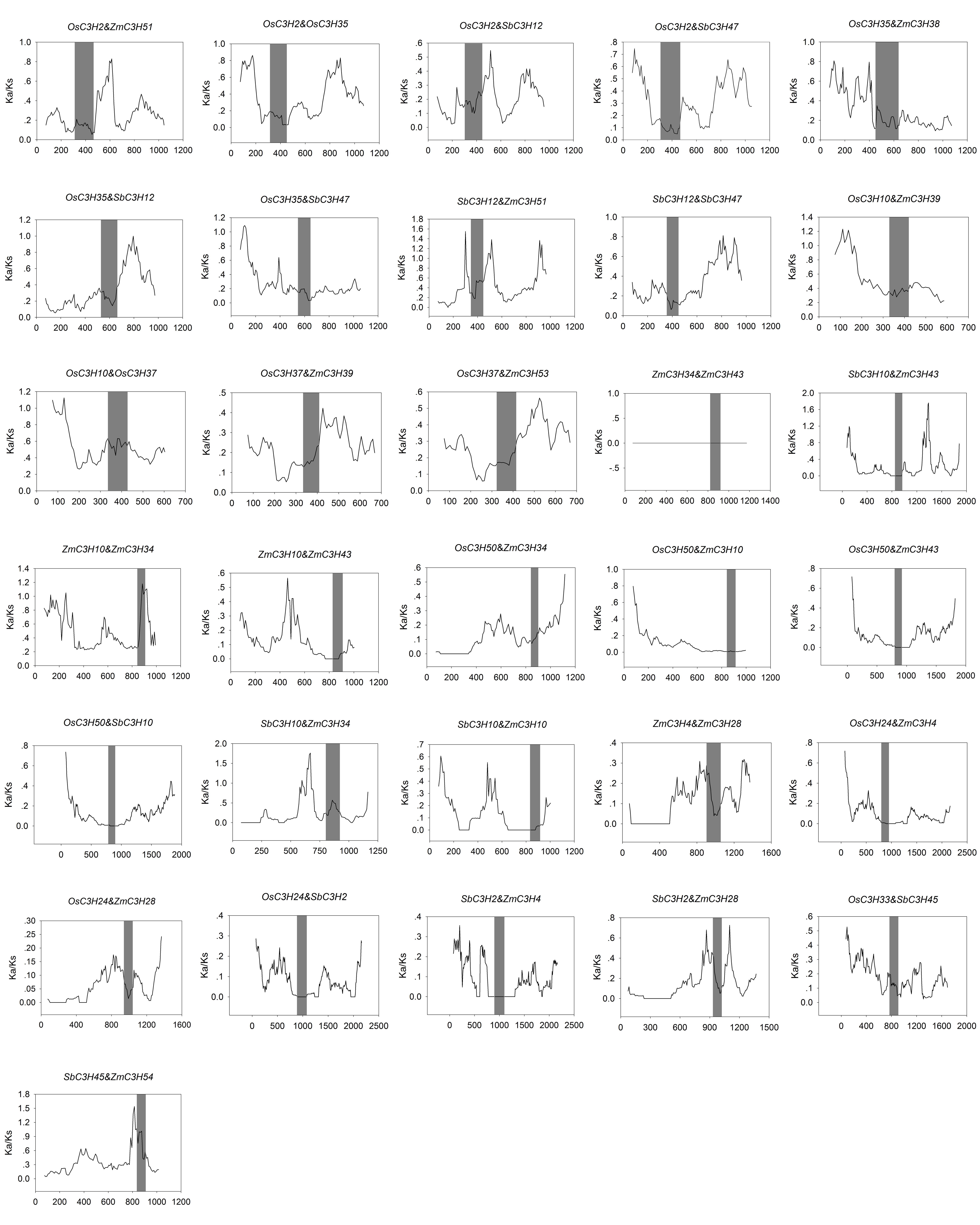

Supplement: Supplementary file 1 — For the big data, we put supplementary figures and tables in Supplementary Material. Supplementary Figure 1 showed expression profiles of CCCH IX genes across different tissues in maize; Supplementary Figure 2 showed phylogenetic relationship of CCCH IX genes constructed by NJ, ML, and MP methods; Supplementary Figure 3 showed sliding window analysis of duplicated CCCH IX genes in three grass species. Supplementary TABLE 3 listed CCCH genes in Sorghum bicolor. Circos use steps: give the detailed steps to draw figure 4 by circos-0.54 program. [file 824287.f1.zip › 824287.f1/figures, tables, and supplementary materials/Figures and Tables/Figures/supplementary figure3.jpg]
