# Supplementary material for: Significant Microsynteny with New Evolutionary Highlights Is Detected through Comparative Genomic Sequence Analysis of Maize CCCH IX Gene Subfamily
Source: Int J Genomics. 2015 Oct 11;2015:824287. doi: 10.1155/2015/824287 (PMC4619961; doi:10.1155/2015/824287)
Supplement: Supplementary file 1 — For the big data, we put supplementary figures and tables in Supplementary Material. Supplementary Figure 1 showed expression profiles of CCCH IX genes across different tissues in maize; Supplementary Figure 2 showed phylogenetic relationship of CCCH IX genes constructed by NJ, ML, and MP methods; Supplementary Figure 3 showed sliding window analysis of duplicated CCCH IX genes in three grass species. Supplementary TABLE 3 listed CCCH genes in Sorghum bicolor. Circos use steps: give the detailed steps to draw figure 4 by circos-0.54 program. [file 824287.f1.zip › 824287.f1/figures, tables, and supplementary materials/Figures and Tables/Tables/table2.doc]

**Table 2** Estimation of the dates of large-scale duplication events in three grasses.

| *Synteny blocks of CCCH IX genes* | *Number of conserved flanking protein-coding genes* | *Synony-mous sites* | *Ks*  *(mean ± s.d.)* | *Date*  *(mya)* |
| --- | --- | --- | --- | --- |
| *OsC3H2&OsC3H35* | 5 | 299.25 | 1.0927±0.4254 | 84.0538 |
| *OsC3H2&SbC3H47* | 3 | 292.50 | 1.0915±0.4868 | 83.9615 |
| *OsC3H2&SbC3H12* | 13 | 268.75 | 0.5470±0.2981 | 42.0769 |
| *OsC3H2&ZmC3H51* | 3 | 293.00 | 0.7827±0.4078 | 60.2077 |
| *OsC3H35&SbC3H12* | 5 | 274.42 | 0.9811±0.3190 | 75.4692 |
| *OsC3H35&SbC3H47* | 3 | 296.67 | 0.4635±0.1314 | 35.6538 |
| *SbC3H12&SbC3H47* | 3 | 271.58 | 0.8632±0.5594 | 66.4000 |
| *SbC3H12&ZmC3H51* | 3 | 275.17 | 0.2552±0.0552 | 19.6308 |
| *OsC3H35&ZmC3H38* | 3 | 302.58 | 0.9811±0.6874 | 75.4692 |
| *OsC3H10&OsC3H37* | 7 | 173.67 | 0.7084±0.2090 | 54.4923 |
| *OsC3H10&ZmC3H39* | 6 | 169.08 | 0.6818±0.1138 | 52.4462 |
| *OsC3H37&ZmC3H39* | 6 | 193.00 | 0.5051±0.0373 | 38.8538 |
| *OsC3H37&ZmC3H53* | 5 | 197.83 | 0.5696±0.1238 | 43.8154 |
| *OsC3H50&ZmC3H10* | 9 | 289.33 | 0.5867±0.1815 | 45.1285 |
| *OsC3H50&SbC3H10* | 16 | 515.00 | 0.5893±0.1738 | 45.3308 |
| *OsC3H50&ZmC3H34* | 5 | 298.42 | 0.6653±0.1309 | 51.1769 |
| *OsC3H50&ZmC3H43* | 6 | 501.00 | 0.6014±0.1371 | 46.2615 |
| *SbC3H10&ZmC3H10* | 7 | 292.67 | 0.1816±0.0469 | 13.9692 |
| *SbC3H10&ZmC3H34* | 5 | 307.92 | 0.1692±0.0579 | 13.0154 |
| *SbC3H10&ZmC3H43* | 6 | 514.33 | 0.1498±0.0200 | 11.5231 |
| *ZmC3H10&ZmC3H34* | 3 | 275.50 | 0.2019±0.0367 | 15.5308 |
| *ZmC3H10&ZmC3H43* | 4 | 291.83 | 0.1990±0.0326 | 15.3077 |
| *ZmC3H34&ZmC3H43* | 5 | 310.50 | 0.0157±0.0352 | 1.2077 |
| *OsC3H24&SbC3H2* | 14 | 572.67 | 0.8338±0.4394 | 64.1385 |
| *OsC3H24&ZmC3H4* | 4 | 571.75 | 0.6299±0.2791 | 48.4539 |
| *OsC3H24&ZmC3H28* | 5 | 359.50 | 0.5979±0.1667 | 45.9923 |
| *SbC3H2&ZmC3H4* | 4 | 570.75 | 0.1853±0.1206 | 14.2538 |
| *SbC3H2&ZmC3H28* | 5 | 359.92 | 0.1921±0.0503 | 14.7769 |
| *ZmC3H4&ZmC3H28* | 4 | 361.00 | 0.2170±0.0754 | 16.6923 |
| *OsC3H33&SbC3H45* | 9 | 458.25 | 0.5963±0.1419 | 45.8692 |
| *SbC3H45&ZmC3H54* | 3 | 269.67 | 0.2567±0.0433 | 19.7462 |
